# Supplementary material for: Automated optimisation of solubility and conformational stability of antibodies and proteins
Source: Nat Commun. 2023 Apr 6;14:1937. doi: 10.1038/s41467-023-37668-6 (PMC10079162; doi:10.1038/s41467-023-37668-6)
Supplement: Supplementary file 3 — Description of Additional Supplementary Files [file 41467_2023_37668_MOESM3_ESM.pdf]

Description of Additional Supplementary Files

Supplementary file 1. Included as a separate pdf file. Final report from the webserver from a run on bacillus licheniformis alpha-amylase (PDB ID 1bli). The report is produced as a html page by the webserver. Page breaks in this and all Supplementary files 2 to 9 below resulted from the conversion to pdf, and may therefore have non-ideal formatting.

Supplementary file 2. Included as a separate pdf file. Final report from the webserver from a run on nanobody Nb.b201 (chain C of PDB ID 5vnw).

Supplementary file 3. Included as a separate pdf file. Final report from the webserver from a run on adalimumab Fab using the post-phase-I MSA with a limit of 5 simultaneous mutations.

Supplementary file 4. Included as a separate pdf file. Final report from the webserver from a run on adalimumab Fab using the OAS human MSA with a limit of 5 simultaneous mutations.

Supplementary file 5. Included as a separate pdf file. Final report from the webserver from a run on golimumab scFv using the OAS human MSA with a limit of 5 simultaneous mutations.

Supplementary file 6. Included as a separate pdf file. Final report from the webserver from a run on CR3022 Fab using the post-phase-I MSA with a limit of 5 simultaneous mutations. PDB ID 6w41 was used as input.

Supplementary file 7. Included as a separate pdf file. Final report from the webserver from a run on CR3022 Fab using the post-phase-I MSA with a limit of 5 simultaneous mutations. PDB ID 7jn5 was used as input. 6w41 differs from 7jn5 in the VH domain, which ends respectively with TVSS and TVVS followed by a different linker to the constant domain.

Supplementary file 8. Included as a separate pdf file. Final report from the webserver from a run on nanobody H11-H4.

Supplementary file 9. Included as a separate pdf file. Final report from the webserver from a run on nanobody H11-D4.

Supplementary file 10. Included as a separate MS Excel file. DMS data used for the analysis reported in Fig. S10. Sheet 1: Columns A to H are from the supplementary materials of Supplementary Ref. [16], and only columns A to C were actually used in this work. Data in rows with a non-empty entry in “Site identified from” (column M) are those plotted in green in Fig. S10, entries flagged as “Shortlisted mutation” (Column N) are those plotted in yellow. Sheet 2 contains some annotation on the processing of the input PDB file and the mapping of their sequences to the sequences used in DMS experiments.
