# Supplementary material for: Automated optimisation of solubility and conformational stability of antibodies and proteins
Source: Nat Commun. 2023 Apr 6;14:1937. doi: 10.1038/s41467-023-37668-6 (PMC10079162; doi:10.1038/s41467-023-37668-6)
Supplement: Supplementary file 4 — Supplementary file 1 [file 41467_2023_37668_MOESM4_ESM.pdf]

CamSol design report for input 1bli

General remarks. This CamSol design procedure is aimed at optimising the solubility of the target protein while retaining or improving its native state stability. This task is achieved by combining four approaches. (1) The CamSol structurally-corrected prediction is carried out to identify potential aggregation hotspots on the surface of the target protein, whose presence may elicit aggregation from the native state. Those residues contributing most to the hotspots aggregation propensity are flagged as candidate mutation sites. (2) The CamSol intrinsic profile is used to identify further mutation sites that contribute strongly to the poor solubility of the unfolded state. In fact, thermal fluctuations may lead to the transient exposure of otherwise buried aggregation-promoting regions leading to aggregation from partially or fully unfolded states. After (1) & (2) the CamSol intrinsic algorithm is used to quickly screen all possible point mutations at these identified sites to create a long-list of candidate mutations that would in principle increase solubility. (3) Sequences homologous to the target protein are automatically identified, and a multiple-sequence alignment (MSA) is performed between them. A position-specific scoring-matrix (PSSM) is calculated from this alignment to identify those residues that are most likely to be found at each position. Mutations to residues more conserved (i.e. with higher PSSM frequency) in the alignments of homologous sequences have been shown to correlate with increased native-state stability (albeit the correlation is not perfect), and more generally mutations to such residues are much better tolerated, and highly unlikely to be deleterious for the native fold. This PSSM is used to filter the long-list of candidate solubilising mutations by selecting only those mutations that increase the frequency at the position under scrutiny. (5) A structure-based atomistic prediction of the stability change upon mutation is carried out with the energy function Fold-X for each shortlisted mutations. Mutations with calculated DDG<0 are predicted to be stabilising. The correlation between measured stability and Fold-X predicted DDG is statistically significant but far from perfect. However, the atomistic calculations of Fold-X are fully independent from, and therefore highly complementary to, the evolutionary frequency difference calculated from the PSSM. Therefore mutations with increased evolutionary frequency and with a negative calculated DDG should be stabilising, or at least should not negatively impact stability. Conversely, the CamSol methods has been shown to be highly quantitative in recapitulating the effect of mutations on measured solubility in different contexts (R~0.9). Consequently mutations that (i) increase the CamSol solubility score, (ii) have a FoldX DDG smaller than 0 and (iii) increase the PSSM frequency are expected to increase protein solubility, while not affecting or even improving native fold stability.

CamSol analysis of input pdb file: 1bli.pdb

These amino acids are excluded from the list of potential substitution targets: C, M

Sequences extracted from input pdb and used for analysis:
lower case residues (if any) are residues of missing coordinates but present in the SEQRES field of the input pdb file. Such residues are considered for the solubility calculation, but are never targeted for mutations and their potential impact on stability is not considered.

```
> 1bli:A
anLNGTLMQYFEWYMPNDGQHWKRLQNDSAYLAEHGITAVWIPPAYKGTSQADVGYGAYDLYDLGEFHQKGTVRTKYGTKGELQSAIKSLHSRDINVYGDVVINHKGGADATEDVTAVEV
DPADRNRVISGEHLIKAWTHFHFPGRGSTYSDFKWHWYHFDGTDWDESRKLNRIYKFQGKAWDWEVSNEFGNYDYLMYADIDYDHPDVAAEIKRWGTWYANELQLDGFRLDAVKHIKFSF
LRDWVNHVREKTGKEMFTVAEYWSYDLGALENYLNKTNFNHSVFDVPLHYQFHAASTQGGGYDMRKLLNGTVVSKHPLKSVTFVDNHDTQPGQSLESTVQTWFKPLAYAFILTRESGYPQ
VFYGDMYGTKGDSQREIPALKHKIEPILKARKQYAYGAQHDYFDHHDIVGWTREGDSSVANSGLAALITDGPGGAKRMYVGRQNAGETWHDITGNRSEPVVINSAGWGEFHVNGGSVSIY
VQR
```

Using log-likelihood pssm. Considering only candidate mutations with positive enrichment (log-likelihood > 0), and further restricting the space of candidate substitutions at each position to those residues that are more likely than the WT one

PSSM used to pick candidate mutations

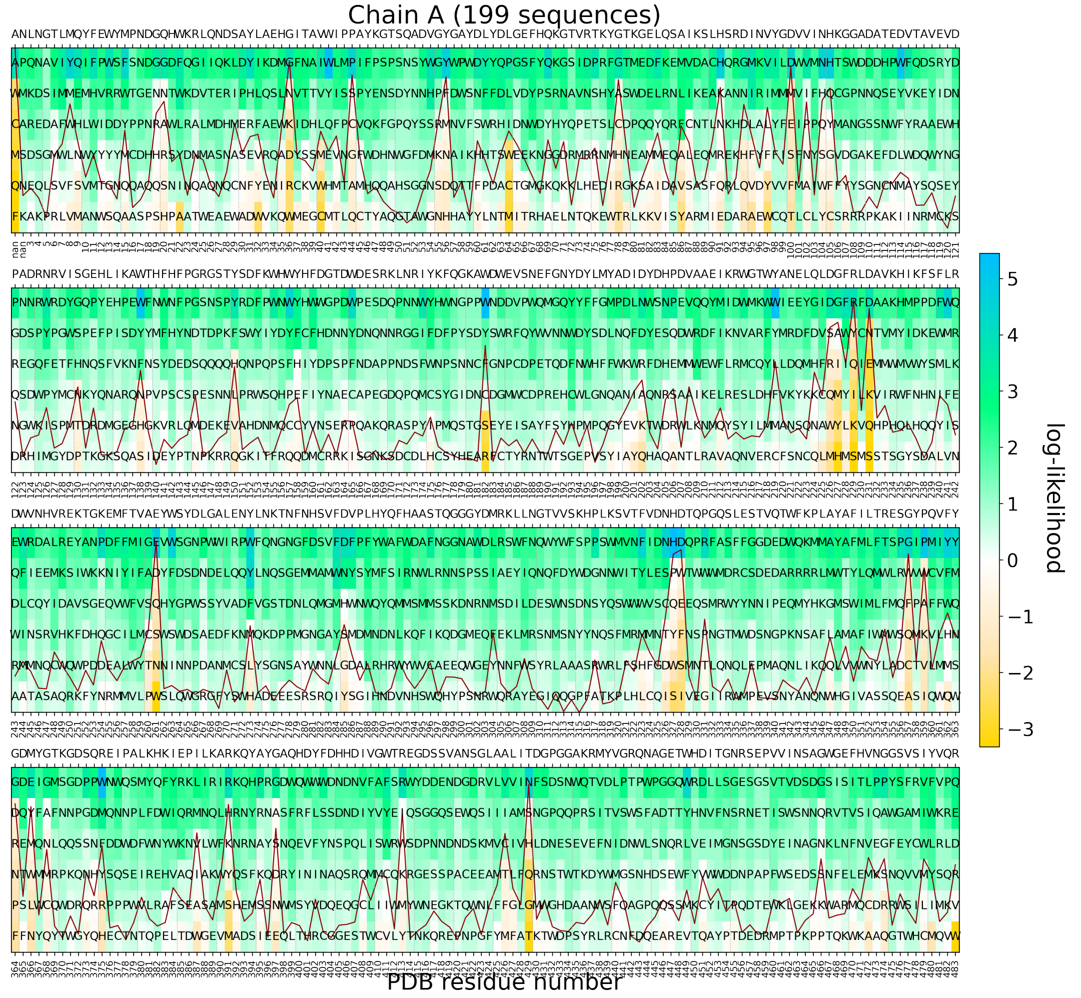

Position-specific scoring matrix (PSSM), as calculated from a multiple-sequence alignment (MSA) of similar sequences. The observed residue frequency (color-bar) is used to select candidate amino acid substitutions. The sequence above the panels is the wild-type (input) sequence as read from the alignment. The red line (if present) is the conservation index of each position (high means position highly conserved). PSSM of chain A read from file hhblits_fullQT_3iteration1kseqs_1bli_chain_A.hhm, computed from 199 aligned sequences (Neff=8).

CamSol intrinsic and structurally corrected profiles

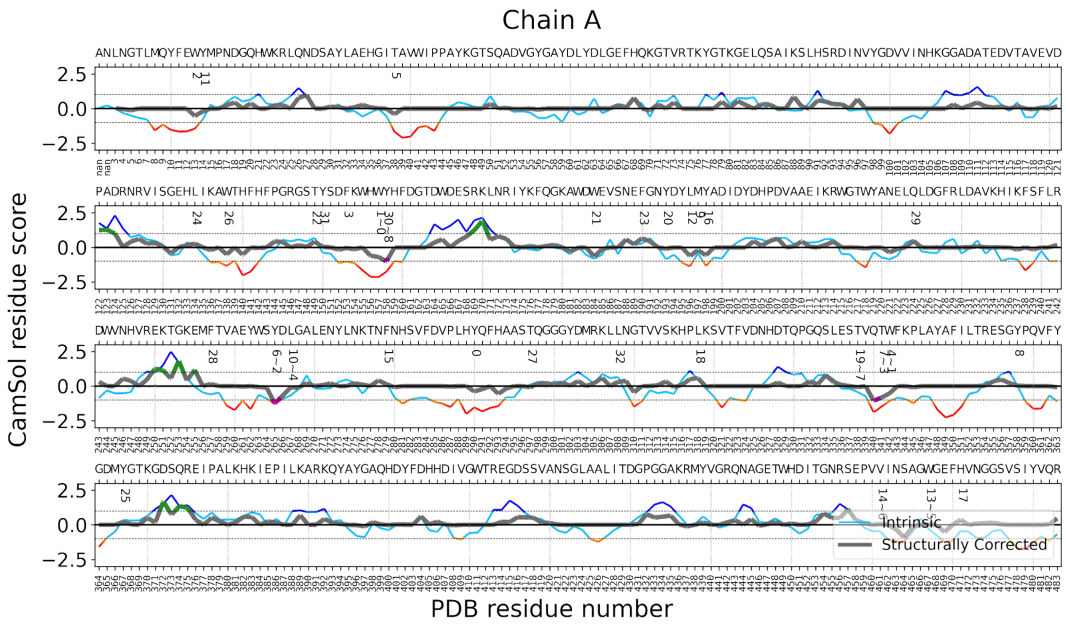

The CamSol intrinsic profile is colour-coded red to blue, where red means aggregation-prone and blue aggregation-resistant. It is common for folded proteins to have large aggregation-prone regions in their intrinsic profile that typically drive the hydrophobic collapse during folding. The structurally corrected profile is color-coded in gray/green/magenta, regions of low negative scores (magenta) are potential aggregation hotspots, regions of high score (green) are solubility promoting. Numbers below the amino acid sequence at the top denote potential mutation sites, identified according to their contribution to the solubility, as well as their accessibility to the solvent.

DESIGN PIPELINE RESULTS: Best Models

Table with identified best combinations of mutations

Design Name	Number of Mutations	Mutations in Combination	Stability Rank	Solubility Rank	Theoretica PI
model_18	9	AA45P,GA81E,NA96R,QA224G,TA338P,TA341P,SA356R,NA473P,GA474P	2	5	6.112
model_7	9	AA45P,GA81E,YA198G,QA224G,TA338P,TA341P,SA356R,NA473P,GA474P	2	4	6.049
model_5	9	AA45P,GA81E,AA209R,QA224G,TA338P,TA341P,SA356R,NA473P,GA474P	2	5	6.112
model_12	8	AA45P,GA81E,QA224G,TA338P,TA341P,SA356R,NA473P,GA474P	3	6	6.049
model_9	8	AA45P,GA81E,NA96R,QA224G,TA338P,TA341P,NA473P,GA474P	4	6	6.049
model_13	8	AA45P,GA81E,NA96R,TA338P,TA341P,SA356R,NA473P,GA474P	3	6	6.112
model_20	6	AA45P,GA81E,TA338P,TA341P,NA473P,GA474P	5	7	5.987
model_22	6	AA45P,GA81E,QA224G,TA338P,TA341P,GA474P	5	8	5.987
model_21	6	AA45P,GA81E,TA338P,TA341P,SA356R,GA474P	5	7	6.049
model_3	2	AA45P,GA81E	10	11	5.987
model_15	2	AA45P,GA474P	10	12	6.047
model_8	2	GA81E,GA474P	10	11	5.987
model_6	1	AA45P	11	12	6.047
model_17	1	GA81E	11	12	5.987
model_1	1	GA474P	11	12	6.047
model_4	12	AA45P,GA81E,NA96R,YA198G,AA209R,QA224G,TA338P,TA341P,SA356R,VA461K,NA473P,GA474P	1	1	6.247
model_11	11	AA45P,GA81E,NA96R,YA198G,AA209R,QA224G,TA338P,TA341P,SA356R,NA473P,GA474P	1	3	6.178
model_2	10	AA45P,GA81E,NA96R,YA198G,QA224G,TA338P,TA341P,SA356R,NA473P,GA474P	2	3	6.112
model_19	7	AA45P,GA81E,QA224G,TA338P,TA341P,NA473P,GA474P	4	7	5.987
model_16	5	AA45P,GA81E,TA338P,TA341P,GA474P	6	8	5.987
model_14	4	AA45P,GA81E,TA341P,GA474P	7	9	5.987
model_10	3	AA45P,GA81E,GA474P	8	11	5.987
WT	0	WT	12	12	6.047

This table contains information on the identified mutation combinations, selected on the basis of their contribution to the overall solubility and stability of the protein. The column 'design name' identifies the pdb file bearing the mutations listed in the column 'mutations in combination'. Combinations belonging to the best combination groups (i.e., combinations containing 1, 2, 6, 8, 9 mutants) are listed first and sorted according to their mutation score. The remaining combinations, not identified as best mutation combinations, are listed afterwards and are sorted according to their mutation score. Mutation combinations are given a solubility and stability rank according to their delta CamSol score and DDG value respectively (i.e. they are binned starting from 1 corresponding the best predicted). The top ranking combination is usually one with the highest 'Number of Mutations'. However, because with each added mutation the chances of introducing a false positive increase, the best combination groups (top rows in the table) embody the best tradeoff between low number of mutations and high gain in solubility and stability. All combinations listed are expected to increase both solubility and stability (or one without compromising the other) and contain single point mutants that are enriched in the input PSSM.

9 Simultaneous Mutations:

Model Name: Model_18

Mutations by chain

Chain A : A45P,G81E,N96R,Q224G,T338P,T341P,S356R,N473P,G474P

Solubility Ranking: 5 | Delta CamSol score: 0.199

Stability Ranking: 2 | FoldX DDG: -15.199 kcal/mol

Mutation Score: 2.239

> Model_18 chain A Optimized_1bli_Repair | A45P G81E N96R Q224G T338P T341P S356R N473P G474P

LNGTLMQYFEWYMPNDGQHWKRLQNDSAYLAEHGITAVWIPP**P**YKGTSQADVGYGAYDLYDLGEFHQKGTVRTKYGTK**E**ELQSAIKSLHSRDI**R**VYGDVVINHKGGADATEDVTAVEVDPA

DRNRVISGEHLIKAWTHFHFPGRGSTYSDFKWHWYHFDGTDWDESRKLNRIYKFQGKAWDWEVSNEFGNYDYLMYADIDYDHPDVAAEIKRWGTWYANEL**G**LDGFRLDAVKHIKFSFLRD

WVNHVREKTGKEMFTVAEYWSYDLGALENYLNKTNFNHSVFDVPLHYQFHAASTQGGGYDMRKLLNGTVVSKHPLKSVTFVDNHDTQPGQSLES**P**VQ**P**WFKPLAYAFILTRE**R**GYPQVFY

GDMYGTKGDSQREIPALKHKIEPILKARKQYAYGAQHDYFDHHDIVGWTREGDSSVANSGLAALITDGPGGAKRMYVGRQNAGETWHDITGNRSEPVVINSAGWGEFHV**PP**GSVSIYVQR

Model Name: Model_7

Mutations by chain

Chain A : A45P,G81E,Y198G,Q224G,T338P,T341P,S356R,N473P,G474P

Solubility Ranking: 4 | Delta CamSol score: 0.242

Stability Ranking: 2 | FoldX DDG: -15.351 kcal/mol

Mutation Score: 2.235

> Model_7 chain A Optimized_1bli_Repair | A45P G81E Y198G Q224G T338P T341P S356R N473P G474P

LNGTLMQYFEWYMPNDGQHWKRLQNDSAYLAEHGITAVWIPP**P**YKGTSQADVGYGAYDLYDLGEFHQKGTVRTKYGTK**E**ELQSAIKSLHSRDINVYGDVVINHKGGADATEDVTAVEVDPA

DRNRVISGEHLIKAWTHFHFPGRGSTYSDFKWHWYHFDGTDWDESRKLNRIYKFQGKAWDWEVSNEFGNYDYLM**G**ADIDYDHPDVAAEIKRWGTWYANEL**G**LDGFRLDAVKHIKFSFLRD

WVNHVREKTGKEMFTVAEYWSYDLGALENYLNKTNFNHSVFDVPLHYQFHAASTQGGGYDMRKLLNGTVVSKHPLKSVTFVDNHDTQPGQSLES**P**VQ**P**WFKPLAYAFILTRE**R**GYPQVFY

GDMYGTKGDSQREIPALKHKIEPILKARKQYAYGAQHDYFDHHDIVGWTREGDSSVANSGLAALITDGPGGAKRMYVGRQNAGETWHDITGNRSEPVVINSAGWGEFHV**PP**GSVSIYVQR

Model Name: Model_5

Mutations by chain

Chain A : A45P,G81E,A209R,Q224G,T338P,T341P,S356R,N473P,G474P

Solubility Ranking: 5 | Delta CamSol score: 0.209

Stability Ranking: 2 | FoldX DDG: -15.156 kcal/mol

Mutation Score: 2.234

> Model_5 chain A Optimized_1bli_Repair | A45P G81E A209R Q224G T338P T341P S356R N473P G474P

LNGTLMQYFEWYMPNDGQHWKRLQNDSAYLAEHGITAVWIPP**P**YKGTSQADVGYGAYDLYDLGEFHQKGTVRTKYGTK**E**ELQSAIKSLHSRDINVYGDVVINHKGGADATEDVTAVEVDPA

DRNRVISGEHLIKAWTHFHFPGRGSTYSDFKWHWYHFDGTDWDESRKLNRIYKFQGKAWDWEVSNEFGNYDYLMYADIDYDHPDV**R**AEIKRWGTWYANEL**G**LDGFRLDAVKHIKFSFLRD

WVNHVREKTGKEMFTVAEYWSYDLGALENYLNKTNFNHSVFDVPLHYQFHAASTQGGGYDMRKLLNGTVVSKHPLKSVTFVDNHDTQPGQSLES**P**VQ**P**WFKPLAYAFILTRE**R**GYPQVFY

GDMYGTKGDSQREIPALKHKIEPILKARKQYAYGAQHDYFDHHDIVGWTREGDSSVANSGLAALITDGPGGAKRMYVGRQNAGETWHDITGNRSEPVVINSAGWGEFHV**PP**GSVSIYVQR

8 Simultaneous Mutations:

Model Name: Model_12

Mutations by chain

Chain A : A45P,G81E,Q224G,T338P,T341P,S356R,N473P,G474P

Solubility Ranking: 6 | Delta CamSol score: 0.188

Stability Ranking: 3 | FoldX DDG: -14.27 kcal/mol

Mutation Score: 2.057

> Model_12 chain A Optimized_1bli_Repair | A45P G81E Q224G T338P T341P S356R N473P G474P

LNGTLMQYFEWYMPNDGQHWKRLQNDSAYLAEHGITAVWIPP**P**YKGTSQADVGYGAYDLYDLGEFHQKGTVRTKYGTK**E**ELQSAIKSLHSRDINVYGDVVINHKGGADATEDVTAVEVDPA

DRNRVISGEHLIKAWTHFHFPGRGSTYSDFKWHWYHFDGTDWDESRKLNRIYKFQGKAWDWEVSNEFGNYDYLMYADIDYDHPDVAAEIKRWGTWYANEL**G**LDGFRLDAVKHIKFSFLRD

WVNHVREKTGKEMFTVAEYWSYDLGALENYLNKTNFNHSVFDVPLHYQFHAASTQGGGYDMRKLLNGTVVSKHPLKSVTFVDNHDTQPGQSLES**P**VQ**P**WFKPLAYAFILTRE**R**GYPQVFY

GDMYGTKGDSQREIPALKHKIEPILKARKQYAYGAQHDYFDHHDIVGWTREGDSSVANSGLAALITDGPGGAKRMYVGRQNAGETWHDITGNRSEPVVINSAGWGEFHV**PP**GSVSIYVQR

Model Name: Model_9

Mutations by chain

Chain A : A45P,G81E,N96R,Q224G,T338P,T341P,N473P,G474P

Solubility Ranking: 6 | Delta CamSol score: 0.173

Stability Ranking: 4 | FoldX DDG: -13.43 kcal/mol

Mutation Score: 2.017

> Model_9 chain A Optimized_1bli_Repair | A45P G81E N96R Q224G T338P T341P N473P G474P

LNGTLMQYFEWYMPNDGQHWKRLQNDSAYLAEHGITAVWIPP**P**YKGTSQADVGYGAYDLYDLGEFHQKGTVRTKYGTK**E**ELQSAIKSLHSRDI**R**VYGDVVINHKGGADATEDVTAVEVDPA

DRNRVISGEHLIKAWTHFHFPGRGSTYSDFKWHWYHFDGTDWDESRKLNRIYKFQGKAWDWEVSNEFGNYDYLMYADIDYDHPDVAAEIKRWGTWYANEL**G**LDGFRLDAVKHIKFSFLRD

WVNHVREKTGKEMFTVAEYWSYDLGALENYLNKTNFNHSVFDVPLHYQFHAASTQGGGYDMRKLLNGTVVSKHPLKSVTFVDNHDTQPGQSLES**P**VQ**P**WFKPLAYAFILTRESGYPQVFY

GDMYGTKGDSQREIPALKHKIEPILKARKQYAYGAQHDYFDHHDIVGWTREGDSSVANSGLAALITDGPGGAKRMYVGRQNAGETWHDITGNRSEPVVINSAGWGEFHV**PP**GSVSIYVQR

Model Name: Model_13

Mutations by chain

Chain A : A45P,G81E,N96R,T338P,T341P,S356R,N473P,G474P

Solubility Ranking: 6 | Delta CamSol score: 0.188

Stability Ranking: 3 | FoldX DDG: -13.763 kcal/mol

Mutation Score: 2.015

> Model_13 chain A Optimized_1bli_Repair | A45P G81E N96R T338P T341P S356R N473P G474P

LNGTLMQYFEWYMPNDGQHWKRLQNDSAYLAEHGITAVWIPP**P**YKGTSQADVGYGAYDLYDLGEFHQKGTVRTKYGTK**E**ELQSAIKSLHSRDI**R**VYGDVVINHKGGADATEDVTAVEVDPA

DRNRVISGEHLIKAWTHFHFPGRGSTYSDFKWHWYHFDGTDWDESRKLNRIYKFQGKAWDWEVSNEFGNYDYLMYADIDYDHPDVAAEIKRWGTWYANELQLDGFRLDAVKHIKFSFLRD

WVNHVREKTGKEMFTVAEYWSYDLGALENYLNKTNFNHSVFDVPLHYQFHAASTQGGGYDMRKLLNGTVVSKHPLKSVTFVDNHDTQPGQSLES**P**VQ**P**WFKPLAYAFILTRE**R**GYPQVFY

GDMYGTKGDSQREIPALKHKIEPILKARKQYAYGAQHDYFDHHDIVGWTREGDSSVANSGLAALITDGPGGAKRMYVGRQNAGETWHDITGNRSEPVVINSAGWGEFHV**PP**GSVSIYVQR

6 Simultaneous Mutations:

Model Name: Model_20

Mutations by chain

Chain A : A45P,G81E,T338P,T341P,N473P,G474P

Solubility Ranking: 7 | Delta CamSol score: 0.151

Stability Ranking: 5 | FoldX DDG: -11.064 kcal/mol

Mutation Score: 1.61

> Model_20 chain A Optimized_1bli_Repair | A45P G81E T338P T341P N473P G474P

LNGTLMQYFEWYMPNDGQHWKRLQNDSAYLAEHGITAVWIPP**P**YKGTSQADVGYGAYDLYDLGEFHQKGTVRTKYGTK**E**ELQSAIKSLHSRDINVYGDVVINHKGGADATEDVTAVEVDPA

DRNRVISGEHLIKAWTHFHFPGRGSTYSDFKWHWYHFDGTDWDESRKLNRIYKFQGKAWDWEVSNEFGNYDYLMYADIDYDHPDVAAEIKRWGTWYANELQLDGFRLDAVKHIKFSFLRD

WVNHVREKTGKEMFTVAEYWSYDLGALENYLNKTNFNHSVFDVPLHYQFHAASTQGGGYDMRKLLNGTVVSKHPLKSVTFVDNHDTQPGQSLES**P**VQ**P**WFKPLAYAFILTRESGYPQVFY

GDMYGTKGDSQREIPALKHKIEPILKARKQYAYGAQHDYFDHHDIVGWTREGDSSVANSGLAALITDGPGGAKRMYVGRQNAGETWHDITGNRSEPVVINSAGWGEFHV**PP**GSVSIYVQR

Model Name: Model_22

Mutations by chain

Chain A : A45P,G81E,Q224G,T338P,T341P,G474P

Solubility Ranking: 8 | Delta CamSol score: 0.126

Stability Ranking: 5 | FoldX DDG: -11.271 kcal/mol

Mutation Score: 1.603

> Model_22 chain A Optimized_1bli_Repair | A45P G81E Q224G T338P T341P G474P

LNGTLMQYFEWYMPNDGQHWKRLQNDSAYLAEHGITAVWIPP**P**YKGTSQADVGYGAYDLYDLGEFHQKGTVRTKYGTK**E**ELQSAIKSLHSRDINVYGDVVINHKGGADATEDVTAVEVDPA

DRNRVISGEHLIKAWTHFHFPGRGSTYSDFKWHWYHFDGTDWDESRKLNRIYKFQGKAWDWEVSNEFGNYDYLMYADIDYDHPDVAAEIKRWGTWYANEL**G**LDGFRLDAVKHIKFSFLRD

WVNHVREKTGKEMFTVAEYWSYDLGALENYLNKTNFNHSVFDVPLHYQFHAASTQGGGYDMRKLLNGTVVSKHPLKSVTFVDNHDTQPGQSLES**P**VQ**P**WFKPLAYAFILTRESGYPQVFY

GDMYGTKGDSQREIPALKHKIEPILKARKQYAYGAQHDYFDHHDIVGWTREGDSSVANSGLAALITDGPGGAKRMYVGRQNAGETWHDITGNRSEPVVINSAGWGEFHVN**P**GSVSIYVQR

Model Name: Model_21

Mutations by chain

Chain A : A45P,G81E,T338P,T341P,S356R,G474P

Solubility Ranking: 7 | Delta CamSol score: 0.141

Stability Ranking: 5 | FoldX DDG: -11.604 kcal/mol

Mutation Score: 1.601

> Model_21 chain A Optimized_1bli_Repair | A45P G81E T338P T341P S356R G474P

LNGTLMQYFEWYMPNDGQHWKRLQNDSAYLAEHGITAVWIPP**P**YKGTSQADVGYGAYDLYDLGEFHQKGTVRTKYGTK**E**ELQSAIKSLHSRDINVYGDVVINHKGGADATEDVTAVEVDPA

DRNRVISGEHLIKAWTHFHFPGRGSTYSDFKWHWYHFDGTDWDESRKLNRIYKFQGKAWDWEVSNEFGNYDYLMYADIDYDHPDVAAEIKRWGTWYANELQLDGFRLDAVKHIKFSFLRD

WVNHVREKTGKEMFTVAEYWSYDLGALENYLNKTNFNHSVFDVPLHYQFHAASTQGGGYDMRKLLNGTVVSKHPLKSVTFVDNHDTQPGQSLES**P**VQ**P**WFKPLAYAFILTRE**R**GYPQVFY

GDMYGTKGDSQREIPALKHKIEPILKARKQYAYGAQHDYFDHHDIVGWTREGDSSVANSGLAALITDGPGGAKRMYVGRQNAGETWHDITGNRSEPVVINSAGWGEFHVN**P**GSVSIYVQR

2 Simultaneous Mutations:

Model Name: Model_3

Mutations by chain

Chain A : A45P,G81E

Solubility Ranking: 11 | Delta CamSol score: 0.038

Stability Ranking: 10 | FoldX DDG: -4.413 kcal/mol

Mutation Score: 0.627

> Model_3 chain A Optimized_1bli_Repair | A45P G81E

LNGTLMQYFEWYMPNDGQHWKRLQNDSAYLAEHGITAVWIPP**P**YKGTSQADVGYGAYDLYDLGEFHQKGTVRTKYGTK**E**ELQSAIKSLHSRDINVYGDVVINHKGGADATEDVTAVEVDPA

DRNRVISGEHLIKAWTHFHFPGRGSTYSDFKWHWYHFDGTDWDESRKLNRIYKFQGKAWDWEVSNEFGNYDYLMYADIDYDHPDVAAEIKRWGTWYANELQLDGFRLDAVKHIKFSFLRD

WVNHVREKTGKEMFTVAEYWSYDLGALENYLNKTNFNHSVFDVPLHYQFHAASTQGGGYDMRKLLNGTVVSKHPLKSVTFVDNHDTQPGQSLESTVQTWFKPLAYAFILTRESGYPQVFY

GDMYGTKGDSQREIPALKHKIEPILKARKQYAYGAQHDYFDHHDIVGWTREGDSSVANSGLAALITDGPGGAKRMYVGRQNAGETWHDITGNRSEPVVINSAGWGEFHVNGGSVSIYVQR

Model Name: Model_15

Mutations by chain

Chain A : A45P,G474P

Solubility Ranking: 12 | Delta CamSol score: 0.023

Stability Ranking: 10 | FoldX DDG: -4.176 kcal/mol

Mutation Score: 0.579

> Model_15 chain A Optimized_1bli_Repair | A45P G474P

LNGTLMQYFEWYMPNDGQHWKRLQNDSAYLAEHGITAVWIPP**P**YKGTSQADVGYGAYDLYDLGEFHQKGTVRTKYGTKGELQSAIKSLHSRDINVYGDVVINHKGGADATEDVTAVEVDPA

DRNRVISGEHLIKAWTHFHFPGRGSTYSDFKWHWYHFDGTDWDESRKLNRIYKFQGKAWDWEVSNEFGNYDYLMYADIDYDHPDVAAEIKRWGTWYANELQLDGFRLDAVKHIKFSFLRD

WVNHVREKTGKEMFTVAEYWSYDLGALENYLNKTNFNHSVFDVPLHYQFHAASTQGGGYDMRKLLNGTVVSKHPLKSVTFVDNHDTQPGQSLESTVQTWFKPLAYAFILTRESGYPQVFY

GDMYGTKGDSQREIPALKHKIEPILKARKQYAYGAQHDYFDHHDIVGWTREGDSSVANSGLAALITDGPGGAKRMYVGRQNAGETWHDITGNRSEPVVINSAGWGEFHVN**P**GSVSIYVQR

Model Name: Model_8

Mutations by chain

Chain A : G81E,G474P

Solubility Ranking: 11 | Delta CamSol score: 0.029

Stability Ranking: 10 | FoldX DDG: -4.144 kcal/mol

Mutation Score: 0.572

> Model_8 chain A Optimized_1bli_Repair | G81E G474P

LNGTLMQYFEWYMPNDGQHWKRLQNDSAYLAEHGITAVWIPPAYKGTSQADVGYGAYDLYDLGEFHQKGTVRTKYGTK**E**ELQSAIKSLHSRDINVYGDVVINHKGGADATEDVTAVEVDPA

DRNRVISGEHLIKAWTHFHFPGRGSTYSDFKWHWYHFDGTDWDESRKLNRIYKFQGKAWDWEVSNEFGNYDYLMYADIDYDHPDVAAEIKRWGTWYANELQLDGFRLDAVKHIKFSFLRD

WVNHVREKTGKEMFTVAEYWSYDLGALENYLNKTNFNHSVFDVPLHYQFHAASTQGGGYDMRKLLNGTVVSKHPLKSVTFVDNHDTQPGQSLESTVQTWFKPLAYAFILTRESGYPQVFY

GDMYGTKGDSQREIPALKHKIEPILKARKQYAYGAQHDYFDHHDIVGWTREGDSSVANSGLAALITDGPGGAKRMYVGRQNAGETWHDITGNRSEPVVINSAGWGEFHVN**P**GSVSIYVQR

1 Single Mutation:

Model Name: Model_6

Mutations by chain

Chain A : A45P

Solubility Ranking: 12 | Delta CamSol score: 0.016

Stability Ranking: 11 | FoldX DDG: -2.222 kcal/mol

Mutation Score: 0.317

> Model_6 chain A Optimized_1bli_Repair | A45P

LNGTLMQYFEWYMPNDGQHWKRLQNDSAYLAEHGITAVWIPPPYKGTSQADVGYGAYDLYDLGEFHQKGTVRTKYGTKGELQSAIKSLHSRDINVYGDVVINHKGGADATEDVTAVEVDPA

DRNRVISGEHLIKAWTHFHFPGRGSTYSDFKWHWYHFDGTDWDESRKLNRIYKFQGKAWDWEVSNEFGNYDYLMYADIDYDHPDVAAEIKRWGTWYANELQLDGFRLDAVKHIKFSFLRD

WVNHVREKTGKEMFTVAEYWSYDLGALENYLNKTNFNHSVFDVPLHYQFHAASTQGGGYDMRKLLNGTVVSKHPLKSVTFVDNHDTQPGQSLESTVQTWFKPLAYAFILTRESGYPQVFY

GDMYGTKGDSQREIPALKHKIEPILKARKQYAYGAQHDYFDHHDIVGWTREGDSSVANSGLAALITDGPGGAKRMYVGRQNAGETWHDITGNRSEPVVINSAGWGEFHVNGGSVSIYVQR

Model Name: Model_17

Mutations by chain

Chain A : G81E

Solubility Ranking: 12 | Delta CamSol score: 0.022

Stability Ranking: 11 | FoldX DDG: -2.19 kcal/mol

Mutation Score: 0.31

> Model_17 chain A Optimized_1bli_Repair | G81E

LNGTLMQYFEWYMPNDGQHWKRLQNDSAYLAEHGITAVWIPPAYKGTSQADVGYGAYDLYDLGEFHQKGTVRTKYGTKEELQSAIKSLHSRDINVYGDVVINHKGGADATEDVTAVEVDPA

DRNRVISGEHLIKAWTHFHFPGRGSTYSDFKWHWYHFDGTDWDESRKLNRIYKFQGKAWDWEVSNEFGNYDYLMYADIDYDHPDVAAEIKRWGTWYANELQLDGFRLDAVKHIKFSFLRD

WVNHVREKTGKEMFTVAEYWSYDLGALENYLNKTNFNHSVFDVPLHYQFHAASTQGGGYDMRKLLNGTVVSKHPLKSVTFVDNHDTQPGQSLESTVQTWFKPLAYAFILTRESGYPQVFY

GDMYGTKGDSQREIPALKHKIEPILKARKQYAYGAQHDYFDHHDIVGWTREGDSSVANSGLAALITDGPGGAKRMYVGRQNAGETWHDITGNRSEPVVINSAGWGEFHVNGGSVSIYVQR

Model Name: Model_1

Mutations by chain

Chain A : G474P

Solubility Ranking: 12 | Delta CamSol score: 0.007

Stability Ranking: 11 | FoldX DDG: -1.953 kcal/mol

Mutation Score: 0.262

> Model_1 chain A Optimized_1bli_Repair | G474P

LNGTLMQYFEWYMPNDGQHWKRLQNDSAYLAEHGITAVWIPPAYKGTSQADVGYGAYDLYDLGEFHQKGTVRTKYGTKGELQSAIKSLHSRDINVYGDVVINHKGGADATEDVTAVEVDPA

DRNRVISGEHLIKAWTHFHFPGRGSTYSDFKWHWYHFDGTDWDESRKLNRIYKFQGKAWDWEVSNEFGNYDYLMYADIDYDHPDVAAEIKRWGTWYANELQLDGFRLDAVKHIKFSFLRD

WVNHVREKTGKEMFTVAEYWSYDLGALENYLNKTNFNHSVFDVPLHYQFHAASTQGGGYDMRKLLNGTVVSKHPLKSVTFVDNHDTQPGQSLESTVQTWFKPLAYAFILTRESGYPQVFY

GDMYGTKGDSQREIPALKHKIEPILKARKQYAYGAQHDYFDHHDIVGWTREGDSSVANSGLAALITDGPGGAKRMYVGRQNAGETWHDITGNRSEPVVINSAGWGEFHVNPGSVSIYVQR

Solubility and Stability enhancing combinations of mutations

In the second part of the pipeline the single-point mutaions are combined to produce combination of mutations that are predicted to enhance both solubility and stability of the input protein. The combination process does not rely on running all the computational methods above mentioned for each possible combination since such a process would be too computationally costly. The single point mutants are combined by means of the sum of two of their main characteristics, namely DDG and Delta frequency. The CamSol score is then computed for each combination. Owing to its high computation speed, re-running the CamSol method for each combination does not slow down the algorithm. Once the three metrics are collected the mutation score for the combinations is calculated. Combinations are being produced until the limit of simultaneously occurring mutations set by the user is reached. Of all combination groups only some are selected as "best" groups. The best combination groups are those ones whose maximum mutation score marks a change in the behavior of the increment of the mutation score thorughout the different combination grous. Such a change in behavior is interpreted as a point in the combination proces from which the contribution of an additional mutant is not as favorable as it has been until that point. For all those groups label as "best" up to 3 combinations are modeled, while for the other groups just one model is returned. In generating the protein model bearing the mutation combination the DDG path of its mutations is checked. For DDG path we mean the contribution that each single mutant in the combination gives, in terms of stability, to the protein. If in applying one mutation after the other we register a non negative DDG value the algorithm tries to correct for it by swapping the single point mutation under scrutiny with another taken from the list of the other mutation combinations of the same combination group and that happens at the same point in the DDG path. If no viable alternative is found after three retries the mutation is simply skipped.

Best mutant combination groups identified for 1,2,6,8,9 simultaneous mutations in combination

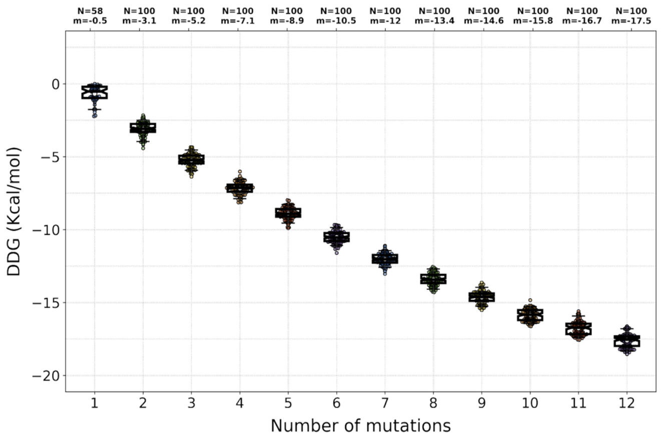

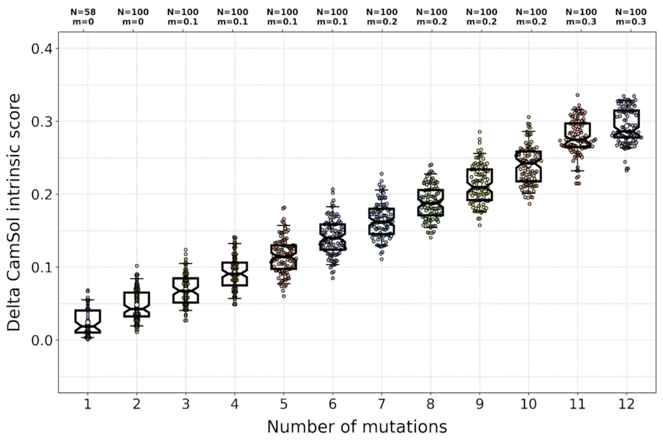

Table with identified candidate mutation sites (105 sites)

Mutation site (seq index)	Mutation site (pdb number)	PSSM score > 0	Delta PSSM score > 0	site conservation	solvent exposure	solubilization potential	Problematic region+site score	region size	residue stru. corr. score	residue intrinsic score	wt residue frequency score	identified from
W156.A	WA157	Y F P		0.403	0.318	0.124	6.671	9	-0.687	-2.155	4.703	Solub. Seq.
W341.A	WA342	R Q K N Y		0.204	0.5	0.106	6.012	13	-0.522	-1.07	2.432	Solub. Seq.
Y264.A	YA265	G S P D N W H	G P S D N H W	0.189	1.0	0.074	3.31	3	-1.254	-1.304	-0.012	Solub. Seq.
T340.A	TA341	D A E P Q N G	D E A P N Q G	0.234	0.58	0.068	6.356	13	-0.828	-1.478	-0.725	Solub. Seq.
L266.A	LA267	P N S A D R E	P N S D E A R	0.189	0.144	0.064	2.612	3	-0.105	-0.678	-0.468	Solub. Seq.
W466.A	WA467	S R K N T Q	S R K N	0.243	0.495	0.06	2.24	3	0.05	-0.278	0.323	Solub. Seq.
V460.A	VA461	T S E W K P	T S E K P W	0.313	0.889	0.058	2.622	3	0.058	-0.465	0.024	Solub. Seq.
T337.A	TA338	G D N S P E R K	D G N S P E R K	0.15	0.22	0.049	5.309	13	-0.075	-0.432	-1.235	Solub. Seq.
Y157.A	YA158	F		0.436	0.606	0.0	6.251	9	-1.02	-1.755	3.263	Solub. Seq.
Y289.A	YA290	W F D H N S Q E	W F	0.162	0.228	0.182	6.63	16	-0.394	-1.531	1.474	Solub. Seq.
W12.A	WA13	R D Y		0.311	0.402	0.123	5.354	8	-0.536	-1.448	3.671	Solub. Seq.

F152.A	FA153	Y P W D I G		0.248	0.228	0.119	5.545	9	-0.048	-1.024	2.739	Solub. Seq.
T37.A	TA38	N D	N	0.422	0.301	0.077	5.865	6	-0.408	-1.67	2.308	Solub. Seq.
Y357.A	YA358	I V P T S	I P V S T	0.324	0.242	0.068	2.643	3	0.024	0.026	0.248	Solub. Seq.
M196.A	MA197	G D F N Q S H	G D N Q S H F	0.212	0.538	0.064	2.298	2	-0.193	-0.433	-1.033	Solub. Seq.
Y13.A	YA14	S R D N Q A	S D R	0.241	0.407	0.064	4.671	8	-0.201	-0.785	0.589	Solub. Seq.
L195.A	LA196	F S H G P V N E	G P S H E N F V	0.129	0.477	0.061	3.247	2	-0.632	-1.37	-0.605	Solub. Seq.
F278.A	FA279	G E N A S Q	G E N S A Q	0.201	0.473	0.054	4.834	16	0.133	0.233	-1.743	Solub. Seq.
Y197.A	YA198	L F Q G	L Q F G	0.326	0.454	0.054	3.158	3	-0.578	-1.344	-0.394	Solub. Seq. & Conservation
H470.A	HA471	T S N E Q K	T E N S K Q	0.318	0.441	0.053	2.383	3	0.361	-0.449	-0.115	Solub. Seq. & Conservation
L317.A	LA318	S N Y Q W T D F E A H K	S N D E K Q Y T A H W F	0.12	1.0	0.052	2.003	5	0.574	0.467	-1.239	Solub. Seq.
Y192.A	YA193	W F H G		0.317	0.16	0.044	1.875	1	-0.105	-0.863	2.816	Solub. Seq.
W183.A	WA184	D N G Y	D	0.244	0.835	0.043	1.869	1	-0.665	-0.824	1.436	Solub. Seq.
T148.A	TA149	P K Q N E R D	P K E R Q N D	0.27	0.582	0.042	3.763	9	0.424	0.688	-0.373	Solub. Seq. & Conservation
F189.A	FA190	V T R W Y L	R T V	0.173	0.671	0.041	0.764	1	0.653	0.271	0.485	Solub. Seq.
L133.A	LA134	E P F N K T G D	E P N K D G T F	0.164	0.814	0.037	3.45	7	0.271	0.181	-1.469	Solub. Seq.
Y366.A	YA367	I F Q W L V	Q I F W	0.257	0.393	0.03	1.881	3	0.203	0.357	0.77	Solub. Seq.
W137.A	WA138	Y F		0.565	0.601	0.021	5.003	7	-0.517	-1.337	5.428	Solub. Seq.
T296.A	TA297	N R S K A Q G	N R S K Q A G	0.159	0.584	0.021	4.901	16	-0.084	0.099	-1.201	Solub. Seq.
F256.A	FA257	Y W I L V		0.301	0.222	0.016	3.715	4	0.148	-0.606	2.674	Solub. Seq.
Q223.A	QA224	G D H	G D H	0.556	0.953	0.015	0.707	2	0.462	0.549	-0.364	Solub. Seq. & Conservation
Y149.A	YA150	F		0.583	0.4	0.0	4.885	9	0.035	-0.389	3.928	Solub. Seq.
L307.A	LA308	F I W Y V	I F	0.335	0.429	0.0	1.297	2	0.307	0.657	1.73	Solub. Seq.
T428.A	TA429	N	N	0.876	0.0	0.023			0.0	-0.065	-3.2	Conservation
S28.A	SA29	L I A	L I A	0.6	0.0	0.0			0.0	0.068	-2.571	Conservation
K105.A	KA106	T V F S I R	T R S V F I	0.309	0.0	0.004			0.0	0.422	-2.941	Conservation

L2.A	LA3	Q K R D E	Q K R D E	0.292	1.0	0.028			0.033	0.009	-2.664	Conservation
G98.A	GA99	L F I V Q	L Q I F V	0.406	0.0	0.0			-0.0	-1.157	-2.215	Conservation
Q19.A	QA20	G	G	0.704	0.529	0.003			0.389	0.657	-1.672	Conservation
R23.A	RA24	G D A N	G D A N	0.452	0.475	0.0			0.254	0.452	-2.004	Conservation
A44.A	AA45	I P V F	P I V F	0.442	0.0	0.016			0.0	0.146	-1.666	Conservation
R374.A	RA375	W F Y	W F Y	0.586	0.839	0.0			1.213	1.415	-1.45	Conservation
A425.A	AA426	V I	V I	0.643	0.0	0.0			-0.0	-1.228	-1.284	Conservation
N95.A	NA96	K R A Y	K R A Y	0.388	0.516	0.011			0.685	-0.081	-1.657	Conservation
A85.A	AA86	L F	L F	0.588	0.0	0.0			0.0	-0.123	-1.345	Conservation
V53.A	VA54	W H S N	W H S N	0.378	0.065	0.006			-0.018	-0.184	-1.603	Conservation
H34.A	HA35	L W A	L A W	0.58	0.127	0.0			0.084	-0.278	-1.293	Conservation
P385.A	PA386	K R N Q E D A	R K N E Q D A	0.294	0.267	0.02			0.165	0.444	-1.704	Conservation
D27.A	DA28	K R H N Q E	K R H N Q E	0.369	0.157	0.009			0.015	0.027	-1.159	Conservation
L224.A	LA225	I V F A	I V F A	0.446	0.0	0.015			-0.0	-0.293	-1.012	Conservation
M365.A	MA366	E Y	E Y	0.771	0.0	0.064			-0.0	-0.48	-0.509	Conservation
H20.A	HA21	D T W S N	D T W S N	0.418	0.0	0.024			0.0	1.063	-0.946	Conservation
G80.A	GA81	E D Q A S	E D Q A S	0.339	0.669	0.022			0.547	0.777	-0.951	Conservation
T111.A	TA112	H S N F	H S N F	0.316	0.297	0.02			0.16	0.948	-0.978	Conservation
A464.A	AA465	D N G S	D N G S	0.262	0.752	0.055			-0.181	-0.512	-0.945	Conservation
S295.A	SA296	F L I W V	F L I W V	0.315	0.0	0.0			-0.0	-0.075	-0.857	Conservation
T5.A	TA6	V I A	V I A	0.449	0.0	0.008			-0.0	-0.674	-0.645	Conservation
E11.A	EA12	P V I Y T	P V I T Y	0.316	0.0	0.0			-0.0	-1.645	-0.835	Conservation
M437.A	MA438	V I F Y W L	V I F Y W L	0.329	0.0	0.0			-0.0	0.029	-0.808	Conservation
A29.A	AA30	D P E	D P E	0.459	0.63	0.011			0.142	-0.008	-0.58	Conservation
A208.A	AA209	Q R W I	R Q W I	0.349	0.169	0.02			0.066	0.243	-0.728	Conservation
V285.A	VA286	F Y W D	F Y D W	0.352	0.0	0.112			-0.0	-1.261	-0.714	Conservation
R353.A	RA354	T W F L S	T S W F L	0.475	0.092	0.0			0.028	0.296	-0.469	Conservation
D113.A	DA114	W Y F L	W Y L F	0.367	0.737	0.0			0.149	0.294	-0.628	Conservation
A389.A	AA390	I L F W V	L I F W V	0.436	0.0	0.0			0.0	0.907	-0.495	Conservation

G215.A	GA216	A L I V Y F	A L I V Y F	0.254	0.0	0.0			0.0	-0.499	-0.752	Conservatio
G409.A	GA410	A V S I	A S V I	0.443	0.0	0.0			-0.0	-0.585	-0.459	Conservatio
N125.A	NA126	W Y F P I	W Y F P I	0.388	0.931	0.0			0.411	0.721	-0.478	Conservatio
V117.A	VA118	S Y A W Q	S A Y W Q	0.27	0.099	0.016			-0.048	-0.625	-0.645	Conservatio
R172.A	RA173	N G S Q P D	N G S P Q D	0.272	0.632	0.0			0.616	0.678	-0.576	Conservatio
E384.A	EA385	R Q K A S	R K Q A S	0.332	0.307	0.01			0.249	-0.051	-0.48	Conservatio
Y9.A	YA10	I L W V A	I A L V W	0.344	0.0	0.067			-0.0	-1.541	-0.442	Conservatio
A424.A	AA425	L I V F	L I V F	0.399	0.0	0.0			-0.0	-1.013	-0.35	Conservatio
L6.A	LA7	I F W V	I F W V	0.501	0.0	0.0			-0.0	-0.781	-0.12	Conservatio
T138.A	TA139	F Y N P	N P F Y	0.275	0.0	0.069			-0.0	-0.949	-0.411	Conservatio
N472.A	NA473	P Q E K D A	P E D K Q A	0.435	0.489	0.041			0.094	-0.951	-0.171	Conservatio
L60.A	LA61	Y F W H P	Y F H W P	0.421	0.0	0.016			-0.0	-0.437	-0.164	Conservatio
T352.A	TA353	F L W Y S A	F L S A W Y	0.324	0.0	0.015			0.0	-0.42	-0.291	Conservatio
T251.A	TA252	A W S D P F E	A S D W P E F	0.279	0.216	0.038			0.535	2.498	-0.357	Conservatio
Q373.A	QA374	P D N	P D N	0.348	1.0	0.038			1.355	1.334	-0.167	Conservatio
T257.A	TA258	I F L W V	L I F W V	0.324	0.0	0.0			0.0	-0.464	-0.198	Conservatio
V244.A	VA245	R I N T K Q W F	R I N K Q T W F	0.275	0.0	0.033			0.0	-0.526	-0.23	Conservatio
G467.A	GA468	I V L F	L I V F	0.256	0.0	0.0			-0.0	-0.741	-0.258	Conservatio
H280.A	HA281	D Q N W S	D N Q S W	0.287	0.0	0.049			-0.0	-1.265	-0.184	Conservatio
S416.A	SA417	D G N E	D N G	0.276	0.929	0.031			0.548	0.823	0.521	Exposed Solub.
S355.A	SA356	P R Q	P R Q	0.384	0.295	0.026			0.475	0.86	0.067	Exposed Solub.
G453.A	GA454	S N D T	S N	0.274	0.59	0.026			0.82	0.79	1.483	Exposed Solub.
D242.A	DA243	E Q W R A K Y N	E Q	0.253	0.33	0.026			0.305	-0.861	1.017	Exposed Solub.
N220.A	NA221	E R D K S T	E R D K	0.269	0.493	0.022			-0.37	-0.867	0.738	Exposed Solub.
Q83.A	QA84	K R E	K R	0.373	0.323	0.021			0.316	-0.05	1.435	Exposed Solub.
Q25.A	QA26	I T A E	I T A	0.305	0.315	0.017			0.775	1.488	0.235	Exposed Solub.

H67.A	HA68	Y P D N G L	Y P D N G	0.267	0.606	0.016			0.776	0.605	0.677	Exposed Solub.
S91.A	SA92	Q A K E R D N	Q K E A R D	0.264	0.639	0.015			0.511	0.357	0.569	Exposed Solub.
S84.A	SA85	E N R Q A D K	E R N Q A	0.26	0.39	0.013			0.291	0.232	0.572	Exposed Solub.
S372.A	SA373	P G N	P G	0.462	0.234	0.01			0.73	2.166	0.405	Exposed Solub.
N26.A	NA27	Q E D S A R K	Q E D S	0.252	0.686	0.01			0.964	0.908	0.959	Exposed Solub.
K135.A	KA136	P S R E Q D	P S	0.29	0.335	0.009			-0.197	-1.084	1.073	Exposed Solub.
G473.A	GA474	P A S	P A	0.523	0.437	0.007			0.121	-0.917	0.664	Exposed Solub.
K391.A	KA392	R N Q		0.365	0.255	0.003			0.427	1.162	2.294	Exposed Solub.
K253.A	KA254	P E Q D	P	0.42	0.329	0.002			0.402	0.579	0.475	Exposed Solub.
V72.A	VA73	I P L	I	0.434	0.364	0.001			0.453	0.953	2.119	Exposed Solub.
S129.A	SA130	G H	G	0.486	0.264	0.0			0.079	0.194	1.632	Exposed Solub.
R482.A	RA483	Q E D	E Q D	0.495	0.433	0.0			0.466	-0.674	0.802	Exposed Solub.
I128.A	IA129	Y W F D N	Y W F D	0.313	0.387	0.0			0.326	0.48	0.097	Exposed Solub.
K87.A	KA88	D N E A R S Q	D	0.302	0.663	0.0			0.872	-0.152	1.332	Exposed Solub.

This table contains information on the identified mutation sites, selected on the basis of their contribution to the overall solubility and their solvent exposure, or on the basis of their conservation in the PSSM. The column 'Problematic region+site score' is an indicator (the higher the more aggregation-promoting) of how much a site is expected to contribute to the aggregation propensity accounting for both its own CamSol score and that of the broader sequence region that contains it. Sites identified from the structurally corrected profile are listed first, then those identified from the intrinsic solubility profile, which are also solvent-exposed but don't have a particularly negative structurally-corrected score, and finally those identified from the Conservation (see column 'identified from'). Mutations sites identified from solubility are ranked according to the column 'solubilization potential', which indicates how much the solubility can be improved by mutating that site according to those mutations allowed by the PSSM. As some problematic sites may be highly conserved, the 'solubilization potential' does not necessarily correlate with 'Problematic region+site score'. The 'sovlent exposure' is a score that ranges from 0 (non-exposed) to 1 (as exposed as in the context of a Gly-AminoAcidUnderScrutiny-Gly 3-peptide in an extended conformation), note that a solvent exposure of 0.5 would already offer the largest area to a potential aggregation partner. Sites identifide from 'Conservation' are those where the WT residue has negative log2(enrichment ratio) score from the input PSSM, and the site is relatively well conserved (conservation index > 0.25). The conservation index ranges in 0 to 1 and indicates how conserved a position in the alignment is (red line in the plot of the PSSM). Additional sites may be identified as sites that are solvent exposed, different from the consensus, and whose solubility can be improved with a substitution to a residue that has higher PSSM frequency than the WT residue (sites labelled as Exposed Solub.).

Results of the single-mutation scanning at all suitable sites

mut_id_seqIndex	mut_id_pdb	Mutation Score	Delta CamSol intrinsic score	delta_frequency	DDG (kcal/mol)	Mutation type	CamSol intrinsic score	Mutation frequency
WT	WT	0	0.0	0.0		n/a	0.129	n/a
LA2R	LA3R	0.325	0.025	4.38	-0.371	Conservation	0.155	1.716
AA29P	AA30P	0.28	0.003	3.004	-0.961	Conservation	0.133	2.424
AA44P	AA45P	0.468	0.016	3.837	-2.222	Conservation	0.145	2.171
HA67P	HA68P	0.195	0.003	1.105	-1.255	Exposed Solub.	0.133	1.782
HA67N	HA68N	0.107	0.003	0.824	-0.543	Exposed Solub.	0.133	1.501
GA80E	GA81E	0.442	0.022	3.356	-2.19	Conservation	0.151	2.405

GA80D	GA81D	0.276	0.019	2.775	-0.906	Conservation	0.148	1.824
QA83K	QA84K	0.169	0.018	1.067	-0.869	Exposed Solub.	0.147	2.502
QA83R	QA84R	0.166	0.021	0.58	-1.108	Exposed Solub.	0.15	2.015
SA84E	SA85E	0.125	0.012	1.315	-0.336	Exposed Solub.	0.142	1.887
SA84R	SA85R	0.107	0.013	0.848	-0.431	Exposed Solub.	0.142	1.42
SA84N	SA85N	0.07	0.003	0.904	-0.132	Exposed Solub.	0.132	1.476
SA91K	SA92K	0.079	0.012	0.815	-0.185	Exposed Solub.	0.141	1.384
SA91E	SA92E	0.057	0.009	0.749	-0.025	Exposed Solub.	0.139	1.318
NA95K	NA96K	0.341	0.006	4.191	-0.835	Conservation	0.136	2.534
NA95R	NA96R	0.334	0.011	3.842	-0.929	Conservation	0.14	2.185
KA105R	KA106R	0.258	0.004	3.068	-0.702	Conservation	0.134	0.127
NA125P	NA126P	0.136	0.0	1.674	-0.356	Conservation	0.13	1.196
TA148K	TA149K	0.174	0.039	1.36	-0.535	Solub. Seq.	0.169	0.987
TA148E	TA149E	0.111	0.035	0.935	-0.195	Solub. Seq.	0.164	0.562
TA148R	TA149R	0.112	0.042	0.731	-0.257	Solub. Seq.	0.172	0.358
TA148N	TA149N	0.084	0.018	0.969	-0.084	Solub. Seq.	0.147	0.596
YA197G	YA198G	0.211	0.054	0.817	-1.081	Solub. Seq.	0.183	0.423
AA208R	AA209R	0.308	0.02	3.318	-0.886	Conservation	0.15	2.59
NA220R	NA221R	0.181	0.02	0.864	-1.097	Exposed Solub.	0.149	1.602
NA220K	NA221K	0.086	0.015	0.462	-0.433	Exposed Solub.	0.145	1.2
QA223G	QA224G	0.358	0.011	3.377	-1.436	Solub. Seq.	0.141	3.013
DA242E	DA243E	0.108	0.004	1.078	-0.394	Exposed Solub.	0.133	2.095
VA244I	VA245I	0.083	0.005	1.099	-0.119	Conservation	0.134	0.869
TA251A	TA252A	0.157	0.005	2.461	-0.05	Conservation	0.134	2.104
YA264P	YA265P	0.146	0.067	1.136	-0.114	Solub. Stru.	0.196	1.124
TA296R	TA297R	0.219	0.021	2.413	-0.537	Solub. Seq.	0.15	1.212
TA296K	TA297K	0.22	0.018	1.92	-0.864	Solub. Seq.	0.148	0.719
LA317D	LA318D	0.143	0.042	1.678	-0.001	Solub. Seq.	0.172	0.439
TA337P	TA338P	0.321	0.025	2.011	-1.76	Solub. Stru.	0.154	0.776
TA337R	TA338R	0.239	0.049	1.438	-1.042	Solub. Stru.	0.178	0.203
TA337K	TA338K	0.157	0.044	1.297	-0.35	Solub. Stru.	0.174	0.062
TA340E	TA341E	0.201	0.068	1.699	-0.307	Solub. Stru.	0.197	0.974
TA340P	TA341P	0.311	0.048	1.541	-1.709	Solub. Stru.	0.177	0.816
TA340Q	TA341Q	0.119	0.028	1.337	-0.114	Solub. Stru.	0.157	0.612
SA355P	SA356P	0.28	0.007	3.135	-0.849	Exposed Solub.	0.136	3.202
SA355R	SA356R	0.259	0.026	0.935	-1.77	Exposed Solub.	0.156	1.002
						Exposed		

SA372P	SA373P	0.203	0.01	3.017	-0.124	Solub.	0.139	3.422
QA373P	QA374P	0.223	0.013	2.943	-0.338	Conservation	0.142	2.776
QA373N	QA374N	0.084	0.012	0.972	-0.133	Conservation	0.141	0.805
SA416N	SA417N	0.081	0.006	1.088	-0.1	Exposed Solub.	0.135	1.609
SA416G	SA417G	0.089	0.002	1.15	-0.179	Exposed Solub.	0.131	1.671
VA460E	VA461E	0.174	0.058	1.072	-0.513	Solub. Stru.	0.188	1.096
VA460K	VA461K	0.173	0.055	0.394	-0.944	Solub. Stru.	0.184	0.418
VA460P	VA461P	0.099	0.05	0.283	-0.322	Solub. Stru.	0.179	0.307
AA464D	AA465D	0.265	0.055	3.177	-0.201	Conservation	0.184	2.232
AA464N	AA465N	0.196	0.027	2.499	-0.192	Conservation	0.156	1.554
HA470E	HA471E	0.157	0.053	0.938	-0.477	Solub. Seq.	0.182	0.823
HA470N	HA471N	0.113	0.031	0.997	-0.219	Solub. Seq.	0.16	0.882
HA470S	HA471S	0.186	0.017	1.103	-1.027	Solub. Seq.	0.146	0.988
HA470K	HA471K	0.155	0.041	0.247	-0.991	Solub. Seq.	0.171	0.132
HA470Q	HA471Q	0.14	0.01	0.644	-0.915	Solub. Seq.	0.14	0.529
NA472P	NA473P	0.375	0.041	3.515	-1.23	Conservation	0.17	3.344
GA473P	GA474P	0.375	0.007	2.863	-1.953	Exposed Solub.	0.137	3.527

This table contains information on all possible single mutations at sites identified on the basis of their contribution to the overall solubility and their solvent exposure, as well as to their conservation in the MSA (see previous table). The column 'Mutation type' describes how a site has been identified: Solub. Stru. denotes that it was a site contributing to poor local solubility in the structurally corrected profile; Solub. seq. is the same but for the sequence-based intrinsic profile; Conservation indicates that the wild-type amino acid had log-likelihood<0 in the PSSM; Exposed Solub. are additional solvent-exposed sites where the WT residue is not strongly conserved (other residues with high PSSM score exist at that position) that may be mutated to further increase solubility (albeit unlike Solub. Seq. and Solub. Stru. these are typically not close to or within candidate aggregation hotspots). The column 'mut_id_seqIndex' contains candidate mutations numbered according to the index of the mutation site along the input sequence (from index 0), while in that 'mut_id_pdb' mutations are numbered according to the residue number in the input pdb file. This table has been filtered to contain only point-mutations predicted to increase both predicted solubility and stability - and if the strict-PSSM cutoff is selected as input - also PSSM frequency (see file 1blisingle_mutation_scanning.csv for a full table with all mutations tested, including those predicted destabilising).

Result of single-mutation scanning at all suitable sites

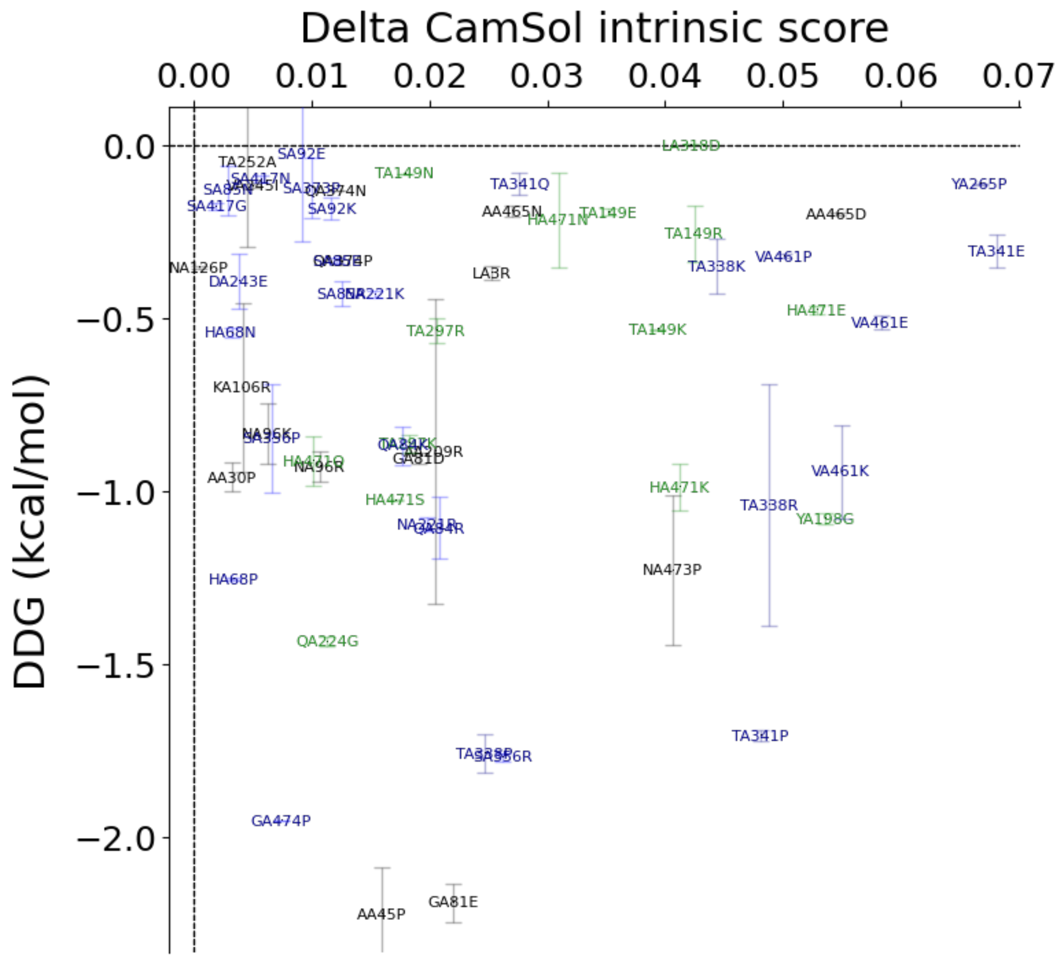

Delta CamSol intrinsic score
0.00
0.01
0.02
0.03
0.04
0.05
0.06
0.07
DDG (kcal/mol)
0.0
−0.5
−1.0
−1.5
−2.0
TA252A
SA92E
SA417N
SA85N
SA417G
TA149N
TA341Q
SA92K
AA465N
HA471N
TA149E
AA465D
YA265P
TA149R
TA338K
VA461P
TA341E
NA126P
DA243E
LA3R
HA68N
TA297R
TA149K
HA471E
VA461E
KA106R
AA30P
NA96R
HA471S
HA471K
TA338R
YA198G
VA461K
NA473P
HA68P
QA224G
TA341P
GA474P
AA45P
GA81E
GA81D

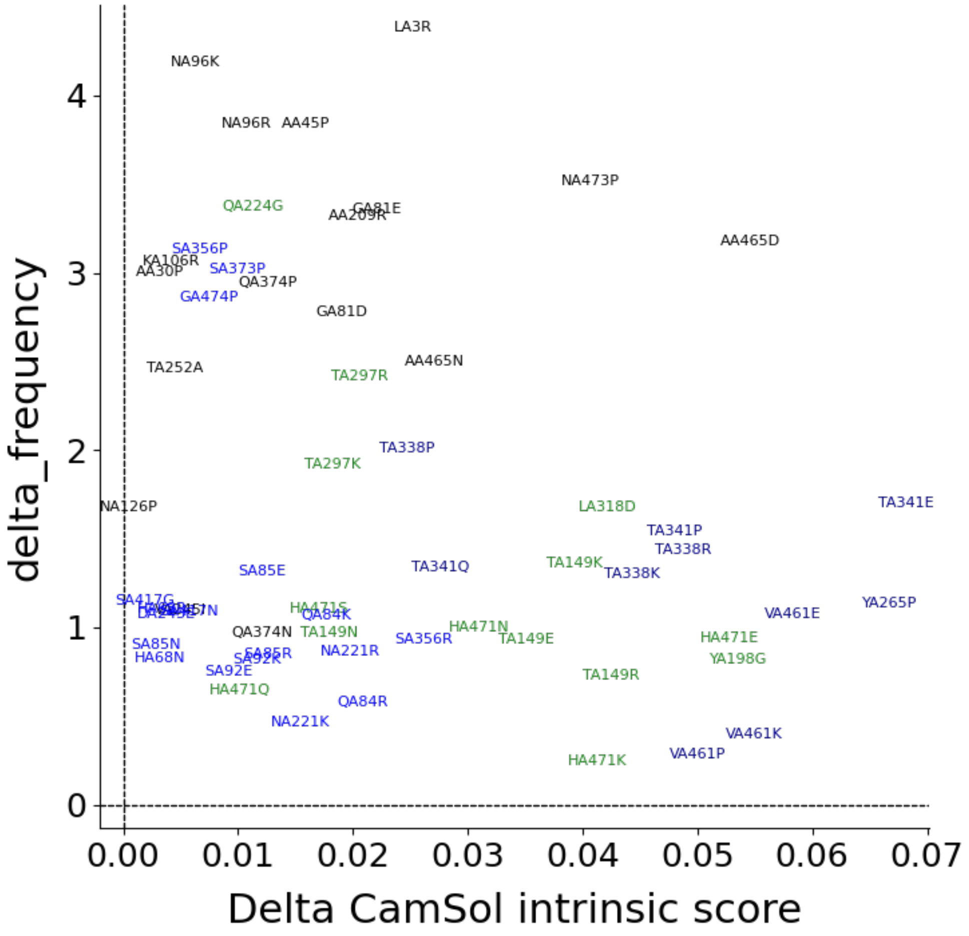

Plot of the data in the previous table, points are candidate mutations numbered according to the residue number within the input pdb file. The best mutations are those with large Delta CamSol score and large negative predicted DDG (if FoldX was run) and/or large positive Delta Frequency in MSA. Points in black correspond to mutation sites selected according to their conservation (see previous table), in green according to the sequence-based intrinsic solubility prediction, and in blue according to the structurally corrected one. Mutations at sites selected according to the solubility profiles (blue and green) are expected to have more impact on solubility than mutations at sites selected from conservation (black).

Mutation score of results of single-mutation scanning at all suitable sites (before normalisation)

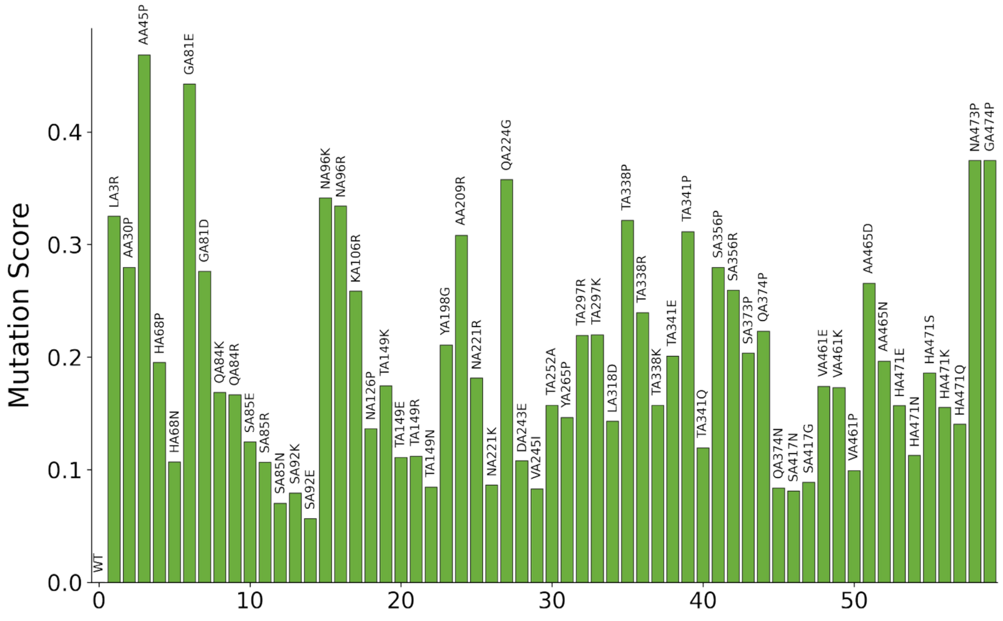

The plotted score is a rather arbitrary combination of delta solubility score, delta frequency, and predicted DDG (if FoldX was run). The highest this score the best the

mutation. This score provides a nice visual ranking of mutations, but in practice one should refer to actual delta solubility score, delta frequency, and predicted DDG values to choose suitable mutations. This is for the single mutational scanning, then for combination of mutations, especially when across multiple chains, the mutation score is normalised usually resulting in a decreased contribution of the PSSM frequency (see paper).
