# Supplementary material for: Automated optimisation of solubility and conformational stability of antibodies and proteins
Source: Nat Commun. 2023 Apr 6;14:1937. doi: 10.1038/s41467-023-37668-6 (PMC10079162; doi:10.1038/s41467-023-37668-6)
Supplement: Supplementary file 7 — Supplementary file 4 [file 41467_2023_37668_MOESM7_ESM.pdf]

CamSol design report for input 3wd5

General remarks. This CamSol design procedure is aimed at optimising the solubility of the target protein while retaining or improving its native state stability. This task is achieved by combining four approaches. (1) The CamSol structurally-corrected prediction is carried out to identify potential aggregation hotspots on the surface of the target protein, whose presence may elicit aggregation from the native state. Those residues contributing most to the hotspots aggregation propensity are flagged as candidate mutation sites. (2) The CamSol intrinsic profile is used to identify further mutation sites that contribute strongly to the poor solubility of the unfolded state. In fact, thermal fluctuations may lead to the transient exposure of otherwise buried aggregation-promoting regions leading to aggregation from partially or fully unfolded states. After (1) & (2) the CamSol intrinsic algorithm is used to quickly screen all possible point mutations at these identified sites to create a long-list of candidate mutations that would in principle increase solubility. (3) Sequences homologous to the target protein are automatically identified, and a multiple-sequence alignment (MSA) is performed between them. A position-specific scoring-matrix (PSSM) is calculated from this alignment to identify those residues that are most likely to be found at each position. Mutations to residues more conserved (i.e. with higher PSSM frequency) in the alignments of homologous sequences have been shown to correlate with increased native-state stability (albeit the correlation is not perfect), and more generally mutations to such residues are much better tolerated, and highly unlikely to be deleterious for the native fold. This PSSM is used to filter the long-list of candidate solubilising mutations by selecting only those mutations that increase the frequency at the position under scrutiny. (5) A structure-based atomistic prediction of the stability change upon mutation is carried out with the energy function Fold-X for each shortlisted mutations. Mutations with calculated DDG<0 are predicted to be stabilising. The correlation between measured stability and Fold-X predicted DDG is statistically significant but far from perfect. However, the atomistic calculations of Fold-X are fully independent from, and therefore highly complementary to, the evolutionary frequency difference calculated from the PSSM. Therefore mutations with increased evolutionary frequency and with a negative calculated DDG should be stabilising, or at least should not negatively impact stability. Conversely, the CamSol methods has been shown to be highly quantitative in recapitulating the effect of mutations on measured solubility in different contexts (R~0.9). Consequently mutations that (i) increase the CamSol solubility score, (ii) have a FoldX DDG smaller than 0 and (iii) increase the PSSM frequency are expected to increase protein solubility, while not affecting or even improving native fold stability.

CamSol analysis of input pdb file: 3wd5.pdb

The following sequence positions are excluded from the design (but their presence is considered in solubility calculations - these may be excluded by the user through the 'Residues that can't be changed' field or becase of e.g. they have missing PSSM information):
Chain A: all
Chain H: A122, S123, T124, K125, G126, P127, S128, V129, F130, P131, L132, A133, P134, S135, S136, K137, S138, T139, S140, G141, G142, T143, A144, A145, L146, G147, C148, L149, V150, K151, D152, Y153, F154, P155, E156, P157, V158, T159, V160, S161, W162, N163, S164, G165, A166, L167, T168, S169, G170, V171, H172, T173, F174, P175, A176, V177, L178, Q179, S180, S181, G182, L183, Y184, S185, L186, S187, S188, V189, V190, T191, V192, P193, S194, S195, S196, L197, G198, T199, Q200, T201, Y202, I203, C204, N205, V206, N207, H208, K209, P210, S211, N212, T213, K214, V215, D216, K217, K218, I219
Chain L: T109, V110, A111, A112, P113, S114, V115, F116, I117, F118, P119, P120, S121, D122, E123, Q124, L125, K126, S127, G128, T129, A130, S131, V132, V133, C134, L135, L136, N137, N138, F139, Y140, P141, R142, E143, A144, K145, V146, Q147, W148, K149, V150, D151, N152, A153, L154, Q155, S156, G157, N158, S159, Q160, E161, S162, V163, T164, E165, Q166, D167, S168, K169, D170, S171, T172, Y173, S174, L175, S176, S177, T178, L179, T180, L181, S182, K183, A184, D185, Y186, E187, K188, H189, K190, V191, Y192, A193, C194, E195, V196, T197, H198, Q199, G200, L201, S202, S203, P204, V205, T206, K207, S208, F209, N210, R211, G212, E213

These amino acids are excluded from the list of potential substitution targets: C, M, N

Sequences extracted from input pdb and used for analysis:
lower case residues (if any) are residues of missing coordinates but present in the SEQRES field of the input pdb file. Such residues are considered for the solubility calculation, but are never targeted for mutations and their potential impact on stability is not considered.

```
> 3wd5:A
vrsssRTPSDKPVAHVVANPQAEGQLQWLNDRANALLANGVELRDNQLVVPSEGLYLIYSQVLFKGQGCPSTHVLLTHTISRIAVSYQTKVNLLSAIKSPCQRETPEGAEAKPWYEPIYL
GGVFQLEKGDRLSAEINRPDYLDFAESGQVYFGIIAL

> 3wd5:H
EVQLVESGGGLVQPGRSLRLSCAASGFTFDDYAMHWVRQAPGKGLEWVSAITWNSGHIDYADSVEGRFTISRDNAKNSLYLDMNSLRAEDTAVYYCAKVSYLSTASSLDYWGQGTLVTVS
SASTKGPSVFPLAPSSkstsgGTAALGCLVKDYFPEPVTVSWNSGALTSGVHTFPAVLQSSGLYSLSSVVTVPSSSLGTQTYICNVNHKPSNTKVDKKI

> 3wd5:L
DIQMTQSPSSLSASVGDRVTITCRASQGIRNYLAWYQQKPGKAPKLLIYAASTLQSGVPSRFSGSGSGTDFTLTISSLQPEDVATYYCQRYNRAPYTFGQGTKVEIKRTVAAPSVFIFPP
SDEQLKSGTASVVCLLNNFYPREAKVQWKVDNALQSGNSQESVTEQDSKDSTYSLSSTLTLSKADYEKHKVYACEVTHQGLSSPVTKSFNRGE
```

Using log-likelihood pssm. Considering only candidate mutations with positive enrichment (log-likelihood > 0), and further restricting the space of candidate substitutions at each position to those residues that are more likely than the WT one

PSSM used to pick candidate mutations

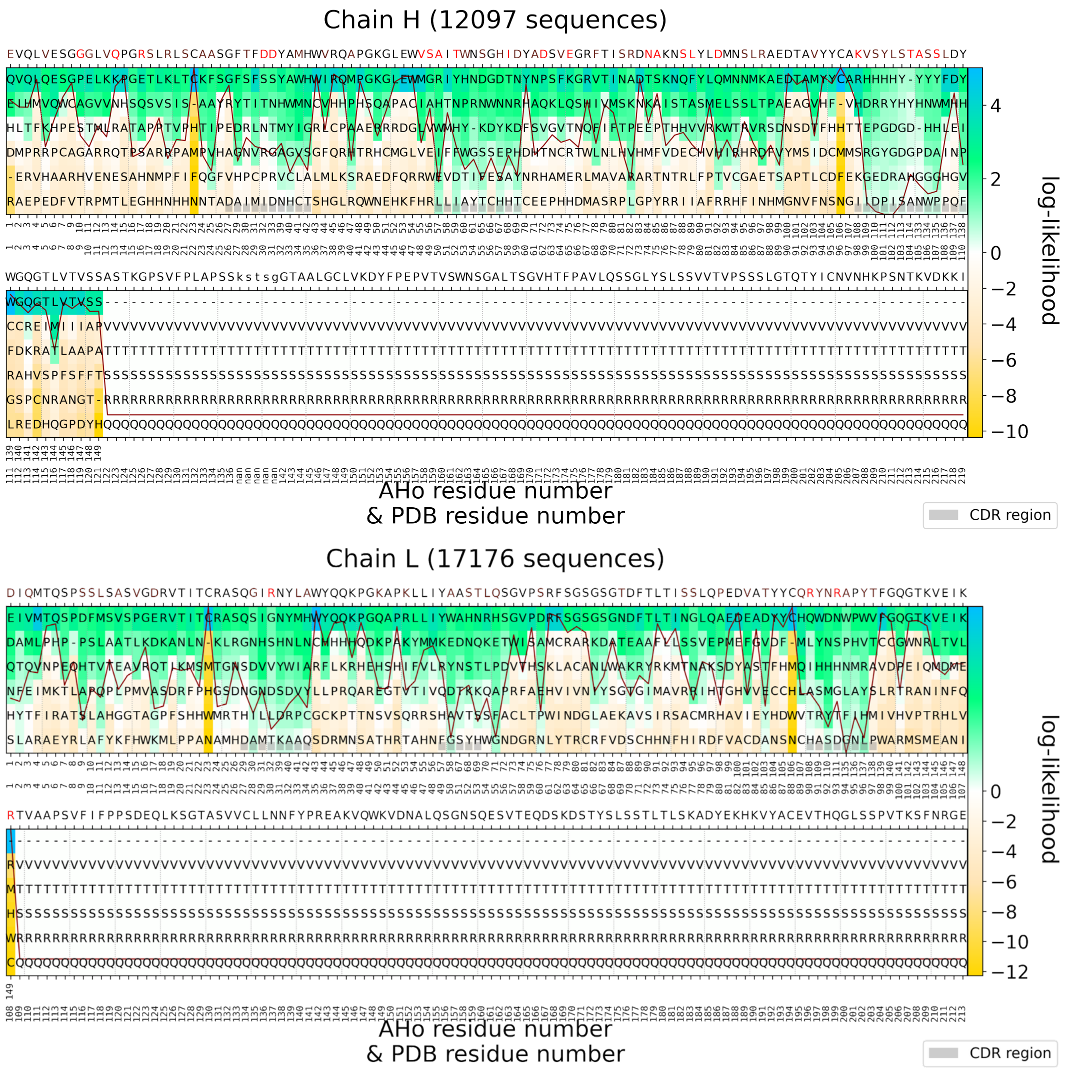

Position-specific scoring matrix (PSSM), as calculated from a multiple-sequence alignment (MSA) of similar sequences. The observed residue frequency (color-bar) is used to select candidate amino acid substitutions. The sequence above the panels is the wild-type (input) sequence as read from the alignment. The red line (if present) is the conservation index of each position (high means position highly conserved). Starting from the top panel: PSSM obtained from MSA of chain H containing 12097 Fv sequences (Fv region only). PSSM obtained from MSA of chain L containing 17176 Fv sequences (Fv region only).

CamSol intrinsic and structurally corrected profiles

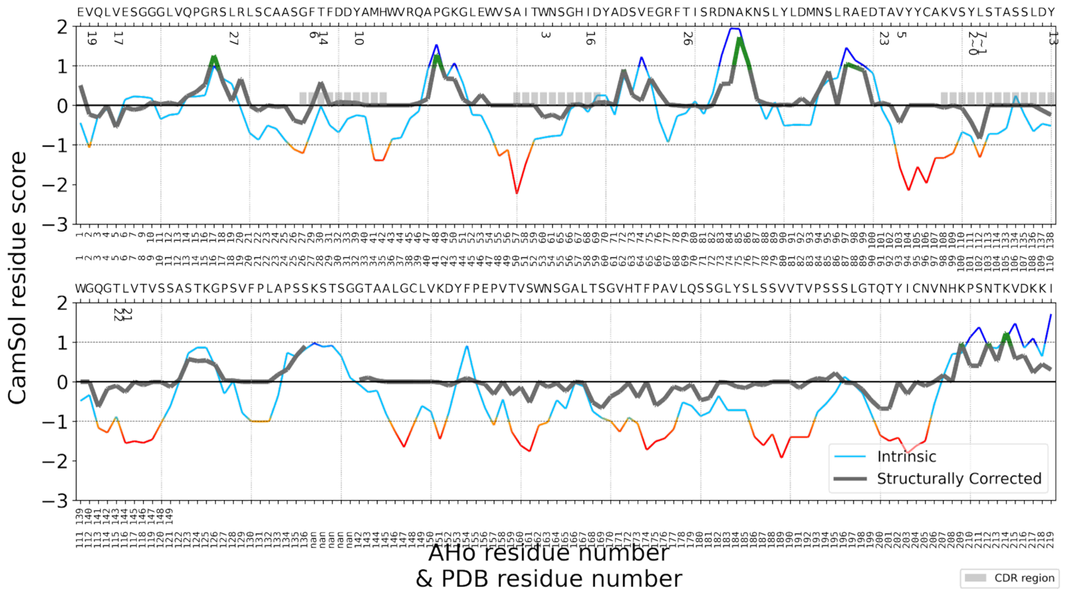

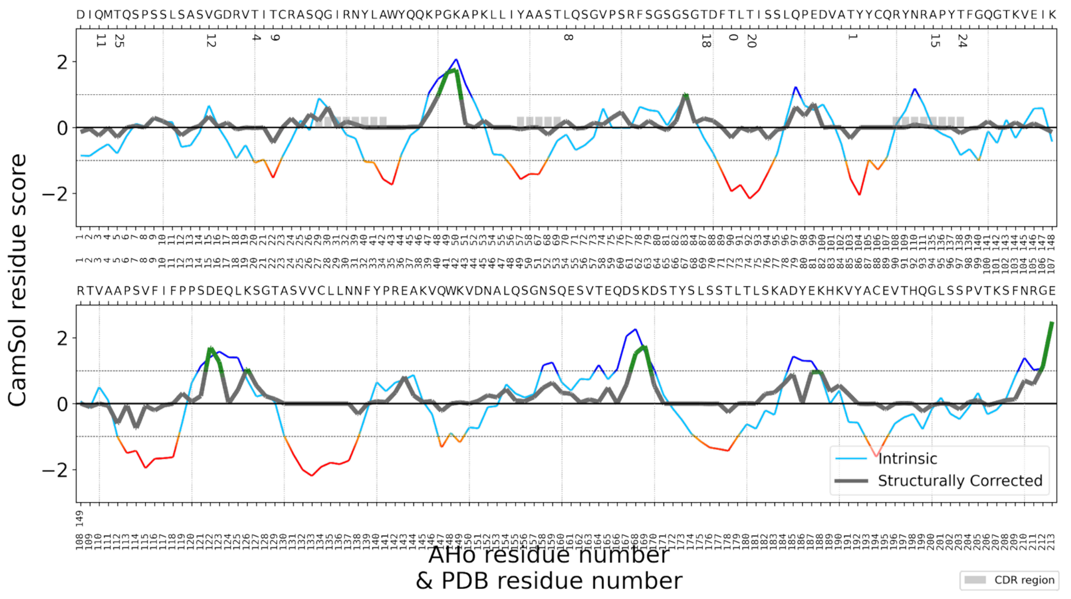

For each chain (see titles of the panels) the CamSol intrinsic profile is colour-coded red to blue, where red means aggregation-prone and blue aggregation-resistant. It is common for folded proteins to have large aggregation-prone regions in their intrinsic profile that typically drive the hydrophobic collapse during folding. The structurally corrected profile is color-coded in gray/green/magenta, regions of low negative scores (magenta) are potential aggregation hotspots, regions of high score (green) are solubility promoting. Numbers below the amino acid sequence at the top denote potential mutation sites, identified according to their contribution to the solubility, as well as their accessibility to the solvent.

DESIGN PIPELINE RESULTS: Best Models

Table with identified best combinations of mutations

Design Name	Number of Mutations	Mutations in Combination	Stability Rank	Solubility Rank	Theoretical PI	Mutation Score
output_4.pdb	2	AH40P,WH53P	3	4	7.955	0.569
output_5.pdb	2	AH40P,AL94P	3	5	7.955	0.555
output_6.pdb	2	AH40P,SH49G	3	6	7.955	0.552
output_1.pdb	1	AH40P	4	6	7.955	0.303
output_2.pdb	1	WH53P	5	4	7.955	0.266
output_3.pdb	1	AL94P	4	6	7.955	0.253
output_12.pdb	5	AH40P,SH49G,WH53P,SH55K,AL94P	1	3	8.187	1.513
output_9.pdb	5	AH23K,AH40P,SH49G,WH53P,AL94P	1	1	8.187	1.255
output_13.pdb	5	AH40P,SH49G,WH53P,AH75S,AL94P	1	3	7.955	1.174
output_8.pdb	4	AH40P,SH49G,WH53P,AL94P	1	3	7.955	1.071
output_7.pdb	3	AH40P,WH53P,AL94P	2	3	7.955	0.822
WT	0	WT	6	6	7.955	0

This table contains information on the identified mutation combinations, selected on the basis of their contribution to the overall solubility and stability of the protein as well as their frequencies in homologous proteins. The column 'design name' identifies the pdb file bearing the mutations listed in the column 'mutations in combination'. Combinations belonging to the best combination groups (i.e., combinations containing 1, 2 mutations) are listed first and sorted according to their mutation score. The remaining combinations, not identified as best mutation combinations, are listed afterwards and are sorted according to their mutation score. Designs are also given a solubility and stability ranking according to their delta CamSol score and DDG value respectively (i.e. they are binned starting from 1 corresponding the best predicted). Note that the mutation score used to rank the designs also depends on residue frequencies in the MSA, and so the correspondence of this ranking with stability and solubility rankings may not be perfect. In general, the top ranking combination is among those with the highest 'Number of Mutations'. However, because with each added mutation the chances of introducing a false positive increase, the best combination groups (top rows in the table) embody the best tradeoff between total number of mutations and high gain in solubility and stability. All combinations listed are expected to increase both solubility and stability (or one without compromising the other) and contain single point mutants that are enriched in the input PSSM. See file Final_table.csv for a more detailed breakdown.

2 Simultaneous Mutations:

Model Name: output_4.pdb

Mutations by chain

Chain H : A40P,W53P

Solubility Ranking: 4 | Delta CamSol score: 0.075

Stability Ranking: 3 | FoldX DDG: -4.75 kcal/mol

Mutation Score: 0.569

```
> output_4.pdb chain A Optimized_3wd5_Repair
RTPSDKPVAHVVANPQAEGQLQWLNDRANALLANGVELRDNQLVVPSEGLYLIYSQVLFKGQGCPSTHVLLTHTISRIAVSYQTKVNLLSAIKSPCQRETPEGAEAKPWYEPIYLGGVFQL
EKGDRLSAEINRPDYLDFAESGQVYFGIIAL
> output_4.pdb chain H Optimized_3wd5_Repair | A40P W53P
EVQLVESGGGLVQPGRSLRLSCAASGFTFDDYAMHWVRQPPGKGLEWVSAITPNSGHIDYADSVEGRFTISRDNAKNSLYLDMNSLRAEDTAVYYCAKVSYLSTASSLDYWGQGTLVTVSS
ASTKGPSVFPLAPSS-----GTAALGCLVKDYFPEPVTVSWNSGALTSGVHTFPAVLQSSGLYSLSSVVTVPSSSLGTQTYICNVNHKPSNTKVDKKI
> output_4.pdb chain L Optimized_3wd5_Repair
DIQMTQSPSSLSASVGDRVTITCRASQGIRNYLAWYQQKPGKAPKLLIYAASTLQSGVPSRFSGSGSGTDFTLTISSLQPEDVATYYCQRYNRAPYTFGQGTKVEIKRTVAAPSVFIFPPS
DEQLKSGTASVVCLLNNFYPREAKVQWKVDNALQSGNSQESVTEQDSKDSTYSLSSTLTLSKADYEKHKVYACEVTHQGLSSPVTKSFNRGE
```

Model Name: output_5.pdb

Mutations by chain

Chain H : A40P

Chain L : A94P

Solubility Ranking: 5 | Delta CamSol score: 0.029

Stability Ranking: 3 | FoldX DDG: -4.981 kcal/mol

Mutation Score: 0.555

```
> output_5.pdb chain A Optimized_3wd5_Repair
RTPSDKPVAHVVANPQAEGQLQWLNDRANALLANGVELRDNQLVVPSEGLYLIYSQVLFKGQGCPSTHVLLTHTISRIAVSYQTKVNLLSAIKSPCQRETPEGAEAKPWYEPIYLGGVFQL
EKGDRLSAEINRPDYLDFAESGQVYFGIIAL
> output_5.pdb chain H Optimized_3wd5_Repair | A40P
EVQLVESGGGLVQPGRSLRLSCAASGFTFDDYAMHWVRQPPGKGLEWVSAITWNSGHIDYADSVEGRFTISRDNAKNSLYLDMNSLRAEDTAVYYCAKVSYLSTASSLDYWGQGTLVTVSS
ASTKGPSVFPLAPSS-----GTAALGCLVKDYFPEPVTVSWNSGALTSGVHTFPAVLQSSGLYSLSSVVTVPSSSLGTQTYICNVNHKPSNTKVDKKI
> output_5.pdb chain L Optimized_3wd5_Repair | A94P
DIQMTQSPSSLSASVGDRVTITCRASQGIRNYLAWYQQKPGKAPKLLIYAASTLQSGVPSRFSGSGSGTDFTLTISSLQPEDVATYYCQRYNRPPYTFGQGTKVEIKRTVAAPSVFIFPPS
DEQLKSGTASVVCLLNNFYPREAKVQWKVDNALQSGNSQESVTEQDSKDSTYSLSSTLTLSKADYEKHKVYACEVTHQGLSSPVTKSFNRGE
```

Model Name: output_6.pdb

Mutations by chain

Chain H : A40P,S49G

Solubility Ranking: 6 | Delta CamSol score: 0.023

Stability Ranking: 3 | FoldX DDG: -4.312 kcal/mol

Mutation Score: 0.552

```
> output_6.pdb chain A Optimized_3wd5_Repair
RTPSDKPVAHVVANPQAEGQLQWLNDRANALLANGVELRDNQLVVPSEGLYLIYSQVLFKGQGCPSTHVLLTHTISRIAVSYQTKVNLLSAIKSPCQRETPEGAEAKPWYEPIYLGGVFQL
EKGDRLSAEINRPDYLDFAESGQVYFGIIAL
> output_6.pdb chain H Optimized_3wd5_Repair | A40P S49G
EVQLVESGGGLVQPGRSLRLSCAASGFTFDDYAMHWVRQPPGKGLEWVGAITWNSGHIDYADSVEGRFTISRDNAKNSLYLDMNSLRAEDTAVYYCAKVSYLSTASSLDYWGQGTLVTVSS
ASTKGPSVFPLAPSS-----GTAALGCLVKDYFPEPVTVSWNSGALTSGVHTFPAVLQSSGLYSLSSVVTVPSSSLGTQTYICNVNHKPSNTKVDKKI
> output_6.pdb chain L Optimized_3wd5_Repair
DIQMTQSPSSLSASVGDRVTITCRASQGIRNYLAWYQQKPGKAPKLLIYAASTLQSGVPSRFSGSGSGTDFTLTISSLQPEDVATYYCQRYNRAPYTFGQGTKVEIKRTVAAPSVFIFPPS
DEQLKSGTASVVCLLNNFYPREAKVQWKVDNALQSGNSQESVTEQDSKDSTYSLSSTLTLSKADYEKHKVYACEVTHQGLSSPVTKSFNRGE
```

1 Single Mutation:

Model Name: output_1.pdb

Mutations by chain

Chain H : A40P

Solubility Ranking: 6 | Delta CamSol score: 0.017

Stability Ranking: 4 | FoldX DDG: -2.778 kcal/mol

Mutation Score: 0.303

> output_1.pdb chain A Optimized_3wd5_Repair

RTPSDKPVAHVVANPQAEGQLQWLNDRANALLANGVELRDNQLVVPSEGLYLIYSQVLFKGQGCPSTHVLLTHTISRIAVSYQTKVNLLSAIKSPCQRETPEGAEAKPWYEPIYLGGVFQL

EKGDRLSAEINRPDYLDFAESGQVYFGIIAL

> output_1.pdb chain H Optimized_3wd5_Repair | A40P

EVQLVESGGGLVQPGRSLRLSCAASGFTFDDYAMHWVRQ**P**PGKGLEWVSAITWNSGHIDYADSVEGRFTISRDNAKNSLYLDMNSLRAEDTAVYYCAKVSYLSTASSLDYWGQGTLVTVSS

ASTKGPSVFPLAPSS-----GTAALGCLVKDYFPEPVTVSWNSGALTSGVHTFPAVLQSSGLYSLSSVVTVPSSSLGTQTYICNVNHKPSNTKVDKKI

> output_1.pdb chain L Optimized_3wd5_Repair

DIQMTQSPSSLSASVGDRVTITCRASQGIRNYLAWYQQKPGKAPKLLIYAASTLQSGVPSRFSGSGSGTDFTLTISSLQPEDVATYYCQRYNRAPYTFGQGTKVEIKRTVAAPSVFIFPPS

DEQLKSGTASVVCLLNNFYPREAKVQWKVDNALQSGNSQESVTEQDSKDSTYSLSSTLTLSKADYEKHKVYACEVTHQGLSSPVTKSFNRGE

Model Name: output_2.pdb

Mutations by chain

Chain H : W53P

Solubility Ranking: 4 | Delta CamSol score: 0.058

Stability Ranking: 5 | FoldX DDG: -1.972 kcal/mol

Mutation Score: 0.266

> output_2.pdb chain A Optimized_3wd5_Repair

RTPSDKPVAHVVANPQAEGQLQWLNDRANALLANGVELRDNQLVVPSEGLYLIYSQVLFKGQGCPSTHVLLTHTISRIAVSYQTKVNLLSAIKSPCQRETPEGAEAKPWYEPIYLGGVFQL

EKGDRLSAEINRPDYLDFAESGQVYFGIIAL

> output_2.pdb chain H Optimized_3wd5_Repair | W53P

EVQLVESGGGLVQPGRSLRLSCAASGFTFDDYAMHWVRQAPGKGLEWVSAIT**P**NSGHIDYADSVEGRFTISRDNAKNSLYLDMNSLRAEDTAVYYCAKVSYLSTASSLDYWGQGTLVTVSS

ASTKGPSVFPLAPSS-----GTAALGCLVKDYFPEPVTVSWNSGALTSGVHTFPAVLQSSGLYSLSSVVTVPSSSLGTQTYICNVNHKPSNTKVDKKI

> output_2.pdb chain L Optimized_3wd5_Repair

DIQMTQSPSSLSASVGDRVTITCRASQGIRNYLAWYQQKPGKAPKLLIYAASTLQSGVPSRFSGSGSGTDFTLTISSLQPEDVATYYCQRYNRAPYTFGQGTKVEIKRTVAAPSVFIFPPS

DEQLKSGTASVVCLLNNFYPREAKVQWKVDNALQSGNSQESVTEQDSKDSTYSLSSTLTLSKADYEKHKVYACEVTHQGLSSPVTKSFNRGE

Model Name: output_3.pdb

Mutations by chain

Chain L : A94P

Solubility Ranking: 6 | Delta CamSol score: 0.012

Stability Ranking: 4 | FoldX DDG: -2.204 kcal/mol

Mutation Score: 0.253

> output_3.pdb chain A Optimized_3wd5_Repair

RTPSDKPVAHVVANPQAEGQLQWLNDRANALLANGVELRDNQLVVPSEGLYLIYSQVLFKGQGCPSTHVLLTHTISRIAVSYQTKVNLLSAIKSPCQRETPEGAEAKPWYEPIYLGGVFQL

EKGDRLSAEINRPDYLDFAESGQVYFGIIAL

> output_3.pdb chain H Optimized_3wd5_Repair

EVQLVESGGGLVQPGRSLRLSCAASGFTFDDYAMHWVRQAPGKGLEWVSAITWNSGHIDYADSVEGRFTISRDNAKNSLYLDMNSLRAEDTAVYYCAKVSYLSTASSLDYWGQGTLVTVSS

ASTKGPSVFPLAPSS-----GTAALGCLVKDYFPEPVTVSWNSGALTSGVHTFPAVLQSSGLYSLSSVVTVPSSSLGTQTYICNVNHKPSNTKVDKKI

> output_3.pdb chain L Optimized_3wd5_Repair | A94P

DIQMTQSPSSLSASVGDRVTITCRASQGIRNYLAWYQQKPGKAPKLLIYAASTLQSGVPSRFSGSGSGTDFTLTISSLQPEDVATYYCQRYNR**P**PYTFGQGTKVEIKRTVAAPSVFIFPPS

DEQLKSGTASVVCLLNNFYPREAKVQWKVDNALQSGNSQESVTEQDSKDSTYSLSSTLTLSKADYEKHKVYACEVTHQGLSSPVTKSFNRGE

5 Simultaneous Mutations:

Model Name: output_12.pdb

Mutations by chain

Chain H : A40P,S49G,W53P,S55K

Chain L : A94P

Solubility Ranking: 3 | Delta CamSol score: 0.095

Stability Ranking: 1 | FoldX DDG: -9.863 kcal/mol

Mutation Score: 1.513

```
> output_12.pdb chain A Optimized_3wd5_Repair
RTPSDKPVAHVVANPQAEGQLQWLNDRANALLANGVELRDNQLVVPSEGLYLIYSQVLFKGQGCPSTHVLLTHTISRIAVSYQTKVNLLSAIKSPCQRETPEGAEAKPWYEPIYLGGVFQL
EKGDRLSAEINRPDYLDFAESGQVYFGIIAL
> output_12.pdb chain H Optimized_3wd5_Repair | A40P S49G W53P S55K
EVQLVESGGGLVQPGRSLRLSCAASGFTFDDYAMHWVRQPPGKGLEWVGAITPNKGHIDYADSVEGRFTISRDNAKNSLYLDMNSLRAEDTAVYYCAKVSYLSTASSLDYWGQGTLVTVSS
ASTKGPSVFPLAPSS-----GTAALGCLVKDYFPEPVTVSWNSGALTSGVHTFPAVLQSSGLYSLSSVVTVPSSSLGTQTYICNVNHKPSNTKVDKKI
> output_12.pdb chain L Optimized_3wd5_Repair | A94P
DIQMTQSPSSLSASVGDRVTITCRASQGIRNYLAWYQQKPGKAPKLLIYAASTLQSGVPSRFSGSGSGTDFTLTISSLQPEDVATYYCQRYNRPPYTFGQGTKVEIKRTVAAPSVFIFPPS
DEQLKSGTASVVCLLNNFYPREAKVQWKVDNALQSGNSQESVTEQDSKDSTYSLSSTLTLSKADYEKHKVYACEVTHQGLSSPVTKSFNRGE
```

Model Name: output_9.pdb

Mutations by chain

Chain H : A23K,A40P,S49G,W53P

Chain L : A94P

Solubility Ranking: 1 | Delta CamSol score: 0.131

Stability Ranking: 1 | FoldX DDG: -9.81 kcal/mol

Mutation Score: 1.255

```
> output_9.pdb chain A Optimized_3wd5_Repair
RTPSDKPVAHVVANPQAEGQLQWLNDRANALLANGVELRDNQLVVPSEGLYLIYSQVLFKGQGCPSTHVLLTHTISRIAVSYQTKVNLLSAIKSPCQRETPEGAEAKPWYEPIYLGGVFQL
EKGDRLSAEINRPDYLDFAESGQVYFGIIAL
> output_9.pdb chain H Optimized_3wd5_Repair | A23K A40P S49G W53P
EVQLVESGGGLVQPGRSLRLSCKASGFTFDDYAMHWVRQPPGKGLEWVGAITPNSGHIDYADSVEGRFTISRDNAKNSLYLDMNSLRAEDTAVYYCAKVSYLSTASSLDYWGQGTLVTVSS
ASTKGPSVFPLAPSS-----GTAALGCLVKDYFPEPVTVSWNSGALTSGVHTFPAVLQSSGLYSLSSVVTVPSSSLGTQTYICNVNHKPSNTKVDKKI
> output_9.pdb chain L Optimized_3wd5_Repair | A94P
DIQMTQSPSSLSASVGDRVTITCRASQGIRNYLAWYQQKPGKAPKLLIYAASTLQSGVPSRFSGSGSGTDFTLTISSLQPEDVATYYCQRYNRPPYTFGQGTKVEIKRTVAAPSVFIFPPS
DEQLKSGTASVVCLLNNFYPREAKVQWKVDNALQSGNSQESVTEQDSKDSTYSLSSTLTLSKADYEKHKVYACEVTHQGLSSPVTKSFNRGE
```

Model Name: output_13.pdb

Mutations by chain

Chain H : A40P,S49G,W53P,A75S

Chain L : A94P

Solubility Ranking: 3 | Delta CamSol score: 0.098

Stability Ranking: 1 | FoldX DDG: -8.991 kcal/mol

Mutation Score: 1.174

```
> output_13.pdb chain A Optimized_3wd5_Repair
RTPSDKPVAHVVANPQAEGQLQWLNDRANALLANGVELRDNQLVVPSEGLYLIYSQVLFKGQGCPSTHVLLTHTISRIAVSYQTKVNLLSAIKSPCQRETPEGAEAKPWYEPIYLGGVFQL
EKGDRLSAEINRPDYLDFAESGQVYFGIIAL
> output_13.pdb chain H Optimized_3wd5_Repair | A40P S49G W53P A75S
EVQLVESGGGLVQPGRSLRLSCAASGFTFDDYAMHWVRQPPGKGLEWVGAITPNSGHIDYADSVEGRFTISRDNSKNSLYLDMNSLRAEDTAVYYCAKVSYLSTASSLDYWGQGTLVTVSS
ASTKGPSVFPLAPSS-----GTAALGCLVKDYFPEPVTVSWNSGALTSGVHTFPAVLQSSGLYSLSSVVTVPSSSLGTQTYICNVNHKPSNTKVDKKI
> output_13.pdb chain L Optimized_3wd5_Repair | A94P
DIQMTQSPSSLSASVGDRVTITCRASQGIRNYLAWYQQKPGKAPKLLIYAASTLQSGVPSRFSGSGSGTDFTLTISSLQPEDVATYYCQRYNRPPYTFGQGTKVEIKRTVAAPSVFIFPPS
DEQLKSGTASVVCLLNNFYPREAKVQWKVDNALQSGNSQESVTEQDSKDSTYSLSSTLTLSKADYEKHKVYACEVTHQGLSSPVTKSFNRGE
```

4 Simultaneous Mutations:

Model Name: output_8.pdb

Mutations by chain

Chain H : A40P,S49G,W53P

Chain L : A94P

Solubility Ranking: 3 | Delta CamSol score: 0.092

Stability Ranking: 1 | FoldX DDG: -8.488 kcal/mol

Mutation Score: 1.071

```
> output_8.pdb chain A Optimized_3wd5_Repair

RTPSDKPVAHVVANPQAEGQLQWLNDRANALLANGVELRDNQLVVPSEGLYLIYSQVLFKGQGCPSTHVLLTHTISRIAVSYQTKVNLLSAIKSPCQRETPEGAEAKPWYEPIYLGGVFQL

EKGDRLSAEINRPDYLDFAESGQVYFGIIAL

> output_8.pdb chain H Optimized_3wd5_Repair | A40P S49G W53P

EVQLVESGGGLVQPGRSLRLSCAASGFTFDDYAMHWVRQPPGKGLEWVGAITPNSGHIDYADSVEGRFTISRDNAKNSLYLDMNSLRAEDTAVYYCAKVSYLSTASSLDYWGQGTLVTVSS

ASTKGPSVFPLAPSS-----GTAALGCLVKDYFPEPVTVSWNSGALTSGVHTFPAVLQSSGLYSLSSVVTVPSSSLGTQTYICNVNHKPSNTKVDKKI

> output_8.pdb chain L Optimized_3wd5_Repair | A94P

DIQMTQSPSSLSASVGDRVTITCRASQGIRNYLAWYQQKPGKAPKLLIYAASTLQSGVPSRFSGSGSGTDFTLTISSLQPEDVATYYCQRYNRPPYTFGQGTKVEIKRTVAAPSVFIFPPS

DEQLKSGTASVVCLLNNFYPREAKVQWKVDNALQSGNSQESVTEQDSKDSTYSLSSTLTLSKADYEKHKVYACEVTHQGLSSPVTKSFNRGE
```

3 Simultaneous Mutations:

Model Name: output_7.pdb

Mutations by chain

Chain H : A40P,W53P

Chain L : A94P

Solubility Ranking: 3 | Delta CamSol score: 0.087

Stability Ranking: 2 | FoldX DDG: -6.954 kcal/mol

Mutation Score: 0.822

```
> output_7.pdb chain A Optimized_3wd5_Repair

RTPSDKPVAHVVANPQAEGQLQWLNDRANALLANGVELRDNQLVVPSEGLYLIYSQVLFKGQGCPSTHVLLTHTISRIAVSYQTKVNLLSAIKSPCQRETPEGAEAKPWYEPIYLGGVFQL

EKGDRLSAEINRPDYLDFAESGQVYFGIIAL

> output_7.pdb chain H Optimized_3wd5_Repair | A40P W53P

EVQLVESGGGLVQPGRSLRLSCAASGFTFDDYAMHWVRQPPGKGLEWVSAITPNSGHIDYADSVEGRFTISRDNAKNSLYLDMNSLRAEDTAVYYCAKVSYLSTASSLDYWGQGTLVTVSS

ASTKGPSVFPLAPSS-----GTAALGCLVKDYFPEPVTVSWNSGALTSGVHTFPAVLQSSGLYSLSSVVTVPSSSLGTQTYICNVNHKPSNTKVDKKI

> output_7.pdb chain L Optimized_3wd5_Repair | A94P

DIQMTQSPSSLSASVGDRVTITCRASQGIRNYLAWYQQKPGKAPKLLIYAASTLQSGVPSRFSGSGSGTDFTLTISSLQPEDVATYYCQRYNRPPYTFGQGTKVEIKRTVAAPSVFIFPPS

DEQLKSGTASVVCLLNNFYPREAKVQWKVDNALQSGNSQESVTEQDSKDSTYSLSSTLTLSKADYEKHKVYACEVTHQGLSSPVTKSFNRGE
```

Solubility and Stability enhancing combinations of mutations

In the second part of the pipeline the single-point mutaions are combined to produce combination of mutations that are predicted to enhance both solubility and stability of the input protein. The combination process does not rely on running all the computational methods above mentioned for each possible combination since such a process would be too computationally costly. The single point mutants are combined by means of the sum of two of their main characteristics, namely DDG and Delta frequency. The CamSol score is then computed for each combination. Owing to its high computation speed, re-running the CamSol method for each combination does not slow down the algorithm. Once the three metrics are collected the mutation score for the combinations is calculated. Combinations are being produced until the limit of simultaneously occurring mutations set by the user is reached. Of all combination groups only some are selected as "best" groups. The best combination groups are those ones whose maximum mutation score marks a change in the behavior of the increment of the mutation score thorughout the different combination grous. Such a change in behavior is interpreted as a point in the combination proces from which the contribution of an additional mutant is not as favorable as it has been until that point. For all those groups label as "best" up to 3 combinations are modeled, while for the other groups just one model is returned. In generating the protein model bearing the mutation combination the DDG path of its mutations is checked. For DDG path we mean the contribution that each single mutant in the combination gives, in terms of stability, to the protein. If in applying one mutation after the other we register a non negative DDG value the algorithm tries to correct for it by swapping the single point mutation under scrutiny with another taken from the list of the other mutation combinations of the same combination group and that happens at the same point in the DDG path. If no viable alternative is found after three retries the mutation is simply skipped.

Best mutant combination groups identified for 1,2 simultaneous mutations in combination

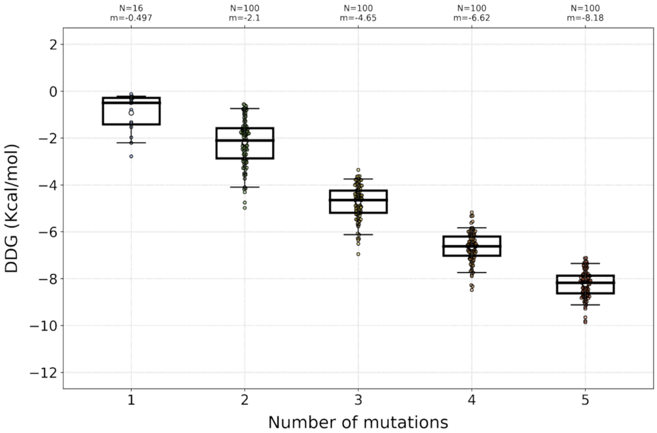

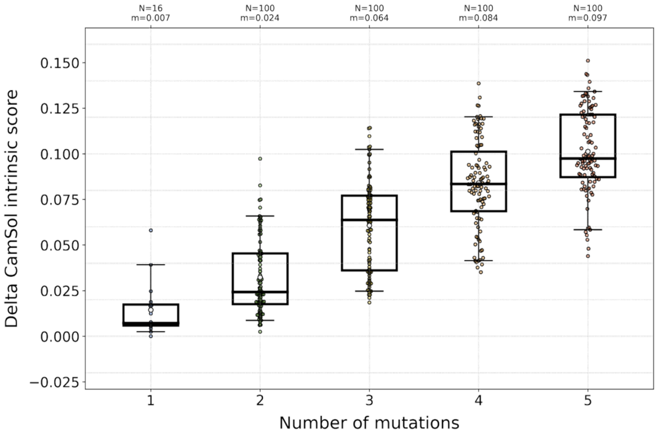

Table with identified candidate mutation sites (69 sites)

Mutation site (seq index)	Mutation site (pdb number)	PSSM score > 0	Delta PSSM score > 0	site conservation	solvent exposure	solubilization potential	Problematic region+site score	region size	residue stru. corr. score	residue intrinsic score	wt residue frequency score	identified from
Y100.H	YH101	H R G D P E F A I	H R G	0.122	0.623	0.063	1.384	1	-0.41	-0.779	0.84	Solub. Stru.
L101.H	LH102	H Y D G R I A F S E P	G D H R Y A P E S I F	0.121	0.696	0.038	1.811	1	-0.851	-1.33	-0.381	Solub. Stru.
T71.L	TL72	A Y I S		0.568	0.344	0.111	6.386	8	-0.31	-1.938	3.267	Solub. Seq.
T84.L	TL85	D V E Y A	D V	0.438	0.256	0.07	4.716	5	-0.331	-1.558	1.684	Solub. Seq.
W52.H	WH53	H P Y T	H P Y	0.395	0.716	0.058	4.33	9	-0.3	-0.814	2.061	Solub. Seq.
T19.L	TL20	K R		0.646	0.606	0.055	3.51	4	-0.013	-1.074	3.434	Solub. Seq.
V92.H	VH93	T I		0.599	0.3	0.043	5.927	7	-0.45	-1.564	3.261	Solub. Seq.
F26.H	FH27	Y D G		0.446	0.195	0.041	2.647	3	-0.001	-0.621	3.187	Solub. Seq.
L53.L	LL54	R K Q S	R K	0.484	0.403	0.032	3.453	7	0.204	-0.209	1.8	Solub. Seq.
T21.L	TL22	S		0.55	0.503	0.023	3.904	4	-0.466	-1.537	3.118	Solub. Seq.
Y31.H	YH32	H A V		0.553	0.269	0.022	2.617	4	0.062	-0.25	3.471	Solub. Seq.
Q2.L	QL3	V E	V	0.64	0.867	0.019	2.039	2	-0.249	-0.667	1.261	Solub. Seq.
V14.L	VL15	P L	P L	0.648	0.435	0.017	0.477	1	0.341	0.679	1.719	Solub. Seq.

Y109.H	YH110	H I P V F		0.425	0.473	0.015	4.356	8	-0.242	-0.517	2.963	Solub. Seq.
T27.H	TH28	S	S	0.589	0.91	0.014	1.968	3	0.6	-0.013	1.785	Solub. Seq.
A93.L	AL94	W P L F G T S H	P W L G T F S H	0.164	0.17	0.012	0.967	1	0.009	-0.014	-0.331	Solub. Seq.
I57.H	IH58	T K P A	T K P A	0.48	0.443	0.012	3.673	9	-0.049	-0.179	-1.477	Solub. Seq. & Conservation
V4.H	VH5	Q K	Q	0.533	0.784	0.008	1.752	1	-0.566	-0.511	2.77	Solub. Seq.
T68.L	TL69	A		0.605	0.452	0.007	4.695	8	0.276	-0.314	3.25	Solub. Seq.
V1.H	VH2	I		0.775	0.283	0.003	2.317	1	-0.23	-1.076	3.675	Solub. Seq.
T73.L	TL74			0.8	0.078	0.0	6.612	8	-0.111	-2.162	3.726	Solub. Seq.
L115.H	LH116	T		0.651	0.229	0.0	5.379	8	-0.265	-1.555	3.53	Solub. Seq.
T114.H	TH115			0.894	0.131	0.0	4.67	8	-0.102	-0.874	3.67	Solub. Seq.
T90.H	TH91			0.912	0.199	0.0	4.402	7	0.066	-0.103	3.697	Solub. Seq.
T96.L	TL97	V	V	0.595	0.28	0.0	1.81	1	-0.17	-0.839	3.007	Solub. Seq.
T4.L	TL5			0.904	0.519	0.0	1.716	1	-0.302	-0.792	3.888	Solub. Seq.
T68.H	TH69	V		0.789	0.657	0.0	1.304	1	-0.022	-0.197	3.504	Solub. Seq.
L17.H	LH18	V		0.708	0.154	0.0	0.459	1	0.096	0.544	3.623	Solub. Seq.
R107.L	RL108			1.019	0.36	0.0			0.005	0.069	-8.654	Conservation
S48.H	SH49	G A	G A	0.768	0.0	0.006			-0.0	-1.12	-5.555	Conservation
S77.H	SH78	Q T H	Q T H	0.62	0.076	0.0			0.043	-0.368	-5.718	Conservation
A49.H	AH50	R H W I E L Y	R E H I W L Y	0.32	0.0	0.041			-0.0	-2.244	-4.762	Conservation
T51.H	TH52	Y H F D I	Y D H F I	0.538	0.0	0.069			-0.0	-0.856	-3.943	Conservation
R15.H	RH16	E Q A	E Q A	0.499	0.939	0.0			1.273	1.007	-3.84	Conservation
D61.H	DH62	P Q V	P Q V	0.54	1.0	0.0			0.91	0.724	-3.588	Conservation
D29.H	DH30	S T	S T	0.52	0.576	0.0			0.086	-0.688	-3.428	Conservation
R89.L	RL90	Q L I A T H	Q A L T H I	0.513	0.0	0.0			-0.0	0.104	-2.968	Conservation
G8.H	GH9	P A	P A	0.601	0.462	0.0			0.08	0.181	-2.771	Conservation
N73.H	NH74	T K E	T K E	0.679	0.344	0.014			0.551	1.946	-2.131	Conservation
D81.H	DH82	Q E K H T	E Q K H T	0.458	0.411	0.0			0.182	-0.494	-2.527	Conservation
L78.H	LH79	F A V	F A V	0.546	0.0	0.003			0.0	0.098	-2.192	Conservation
A74.H	AH75	S	S	0.857	1.0	0.006			1.738	1.934	-1.513	Conservation
K97.H	KH98	R H	R H	0.77	0.041	0.012			-0.059	-1.326	-1.66	Conservation
Q12.H	QH13	K	K	0.902	0.686	0.007			0.231	0.224	-1.046	Conservation
V47.H	VH48	I L	I L	0.558	0.0	0.024			-0.0	-1.282	-1.593	Conservation
R92.L	RL93	S H G T D	S H G D T	0.466	0.064	0.0			0.041	0.685	-1.366	Conservation
S106.H	SH107	Y W H A G P	H A G Y P W	0.262	0.0	0.004			-0.0	-0.242	-1.423	Conservation
T103.H	TH104	Y D G H A	D G H A Y	0.364	0.0	0.028			-0.0	-0.719	-1.116	Conservation
D30.H	DH31	S T G	S T G	0.487	0.769	0.0			0.072	-0.339	-0.58	Conservation

E64.H	EH65	K Q T R	K Q R T	0.578	0.879	0.004			0.689	0.637	-0.311	Conservatio
R29.L	RL30	G S V D L T	G S D L T V	0.303	0.276	0.0			0.184	-0.223	-0.595	Conservatio
A104.H	AH105	Y H P G R	H Y P R G	0.302	0.0	0.025			-0.0	-0.577	-0.485	Conservatio
S76.L	SL77	G R	G	0.473	0.331	0.044			-0.07	-0.8	2.2	Exposed Solub.
Q78.L	QL79	E K		0.672	0.285	0.043			0.631	1.261	3.648	Exposed Solub.
A22.H	AH23	K T	K	0.48	0.375	0.039			-0.032	-0.592	2.206	Exposed Solub.
N53.H	NH54	R G I		0.457	0.462	0.026			-0.233	-0.776	2.46	Exposed Solub.
S54.H	SH55	D K F T	D K	0.332	0.373	0.021			-0.344	-0.759	1.132	Exposed Solub.
A87.H	AH88	P S		0.542	0.519	0.017			0.966	1.138	3.303	Exposed Solub.
A39.H	AH40	P H S	P	0.451	0.144	0.017			0.163	0.897	2.548	Exposed Solub.
H56.H	HH57	D Y E S	D E Y S	0.327	0.154	0.014			0.026	0.072	0.537	Exposed Solub.
Q54.L	QL55	H E P A F G I V	H E P A G F I	0.257	0.175	0.013			-0.029	-0.685	0.685	Exposed Solub.
R86.H	RH87	K T D	K T	0.458	0.406	0.011			1.051	1.466	2.363	Exposed Solub.
K44.L	KL45	R I V Q	R	0.531	0.579	0.007			0.214	0.127	3.711	Exposed Solub.
S59.L	SL60	D A E P	D A	0.407	0.732	0.007			0.469	-0.003	1.589	Exposed Solub.
Q99.L	QL100	G P T		0.529	0.444	0.006			0.194	-0.102	2.829	Exposed Solub.
D0.L	DL1	E Q	E	0.483	0.441	0.005			-0.135	-0.851	2.995	Exposed Solub.
D16.L	DL17	E Q A G K	E	0.422	0.406	0.003			0.149	-0.412	2.725	Exposed Solub.
S8.L	SL9	D P H A	D P H A	0.355	0.553	0.002			0.293	0.266	0.852	Exposed Solub.
G27.L	GL28	S D I A	S D	0.395	0.465	0.001			0.627	0.588	0.888	Exposed Solub.

This table contains information on the identified mutation sites, selected on the basis of their contribution to the overall solubility and their solvent exposure, or on the basis of their conservation in the PSSM. The column 'Problematic region+site score' is an indicator (the higher the more aggregation-promoting) of how much a site is expected to contribute to the aggregation propensity accounting for both its own CamSol score and that of the broader sequence region that contains it. Sites identified from the structurally corrected profile are listed first, then those identified from the intrinsic solubility profile, which are also solvent-exposed but don't have a particularly negative structurally-corrected score, and finally those identified from the Conservation (see column 'identified from'). Mutations sites identified from solubility are ranked according to the column 'solubilization potential', which indicates how much the solubility can be improved by mutating that site according to those mutations allowed by the PSSM. As some problematic sites may be highly conserved, the 'solubilization potential' does not necessarily correlate with 'Problematic region+site score'. The 'sovlent exposure' is a score that ranges from 0 (non-exposed) to 1 (as exposed as in the context of a Gly-AminoAcidUnderScrutiny-Gly 3-peptide in an extended conformation), note that a solvent exposure of 0.5 would already offer the largest area to a potential aggregation partner. Sites identifide from 'Conservation' are those where the WT residue has negative log2(enrichment ratio) score from the input PSSM, and the site is relatively well conserved (conservation index > 0.25). The conservation index ranges in 0 to 1 and indicates how conserved a position in the alignment is (red line in the plot of the PSSM). Additional sites may be identified as sites that are solvent exposed, different from the consensus, and whose solubility can be improved with a substitution to a residue that has higher PSSM frequency than the WT residue (sites labelled as Exposed Solub.).

Results of the single-mutation scanning at all suitable sites

mut_id_seqIndex	mut_id_pdb	Mutation Score	Delta CamSol intrinsic score	delta_frequency	DDG (kcal/mol)	Mutation type	CamSol intrinsic score	Mutation frequency	Chain A CamSol intrinsic score	Chain H CamSol intrinsic score	[illegible]

WT	WT	0	0.0	0.0		n/a	-0.928	n/a	0.415	-0.055	[illegible]
QH12K	QH13K	0.384	0.007	5.72	-0.34	Conservation	-0.921	4.674	0.415	-0.044	[illegible]
AH22K	AH23K	0.246	0.039	1.248	-1.322	Exposed Solub.	-0.889	3.454	0.415	0.01	[illegible]
AH39P	AH40P	0.343	0.017	0.803	-2.778	Exposed Solub.	-0.911	3.35	0.415	-0.027	[illegible]
SH48G	SH49G	0.697	0.006	8.952	-1.534	Conservation	-0.922	3.397	0.415	-0.045	[illegible]
WH52P	WH53P	0.321	0.058	1.09	-1.972	Solub. Seq.	-0.87	3.15	0.415	0.042	[illegible]
SH54K	SH55K	0.21	0.019	0.895	-1.375	Exposed Solub.	-0.909	2.027	0.415	-0.024	[illegible]
HH56S	HH57S	0.048	0.006	0.261	-0.266	Exposed Solub.	-0.922	0.798	0.415	-0.045	[illegible]
AH74S	AH75S	0.335	0.006	4.648	-0.502	Conservation	-0.922	3.135	0.415	-0.045	[illegible]
LH101R	LH102R	0.109	0.025	0.996	-0.245	Solub. Stru.	-0.903	0.615	0.415	-0.014	[illegible]
TH103A	TH104A	0.088	0.008	1.134	-0.121	Conservation	-0.92	0.017	0.415	-0.042	[illegible]
SH106P	SH107P	0.146	0.004	2.005	-0.218	Conservation	-0.924	0.582	0.415	-0.048	[illegible]
SL8P	SL9P	0.153	0.0	1.826	-0.431	Exposed Solub.	-0.928	2.677	0.415	-0.055	[illegible]
VL14P	VL15P	0.197	0.017	2.181	-0.493	Solub. Seq.	-0.911	3.899	0.415	-0.055	[illegible]
VL14L	VL15L	0.023	0.007	0.04	-0.132	Solub. Seq.	-0.921	1.758	0.415	-0.055	[illegible]
DL16E	DL17E	0.146	0.003	1.076	-0.791	Exposed Solub.	-0.926	3.802	0.415	-0.055	[illegible]
SL59D	SL60D	0.145	0.005	1.833	-0.292	Exposed Solub.	-0.923	3.422	0.415	-0.055	[illegible]
AL93P	AL94P	0.345	0.012	1.876	-2.204	Solub. Seq.	-0.916	1.545	0.415	-0.055	[illegible]

This table contains information on all possible single mutations at sites identified on the basis of their contribution to the overall solubility and their solvent exposure, as well as to their conservation in the MSA (see previous table). The column 'Mutation type' describes how a site has been identified: Solub. Stru. denotes that it was a site contributing to poor local solubility in the structurally corrected profile; Solub. seq. is the same but for the sequence-based intrinsic profile; Conservation indicates that the wild-type amino acid had log-likelihood<0 in the PSSM; Exposed Solub. are additional solvent-exposed sites where the WT residue is not strongly conserved (other residues with high PSSM score exist at that position) that may be mutated to further increase solubility (albeit unlike Solub. Seq. and Solub. Stru. these are typically not close to or within candidate aggregation hotspots). The column 'mut_id_seqIndex' contains candidate mutations numbered according to the index of the mutation site along the input sequence (from index 0), while in that 'mut_id_pdb' mutations are numbered according to the residue number in the input pdb file. This table has been filtered to contain only point-mutations predicted to increase both predicted solubility and stability - and if the strict-PSSM cutoff is selected as input - also PSSM frequency (see file 3wd5single_mutation_scanning.csv for a full table with all mutations tested, including those predicted destabilising).

Result of single-mutation scanning at all suitable sites

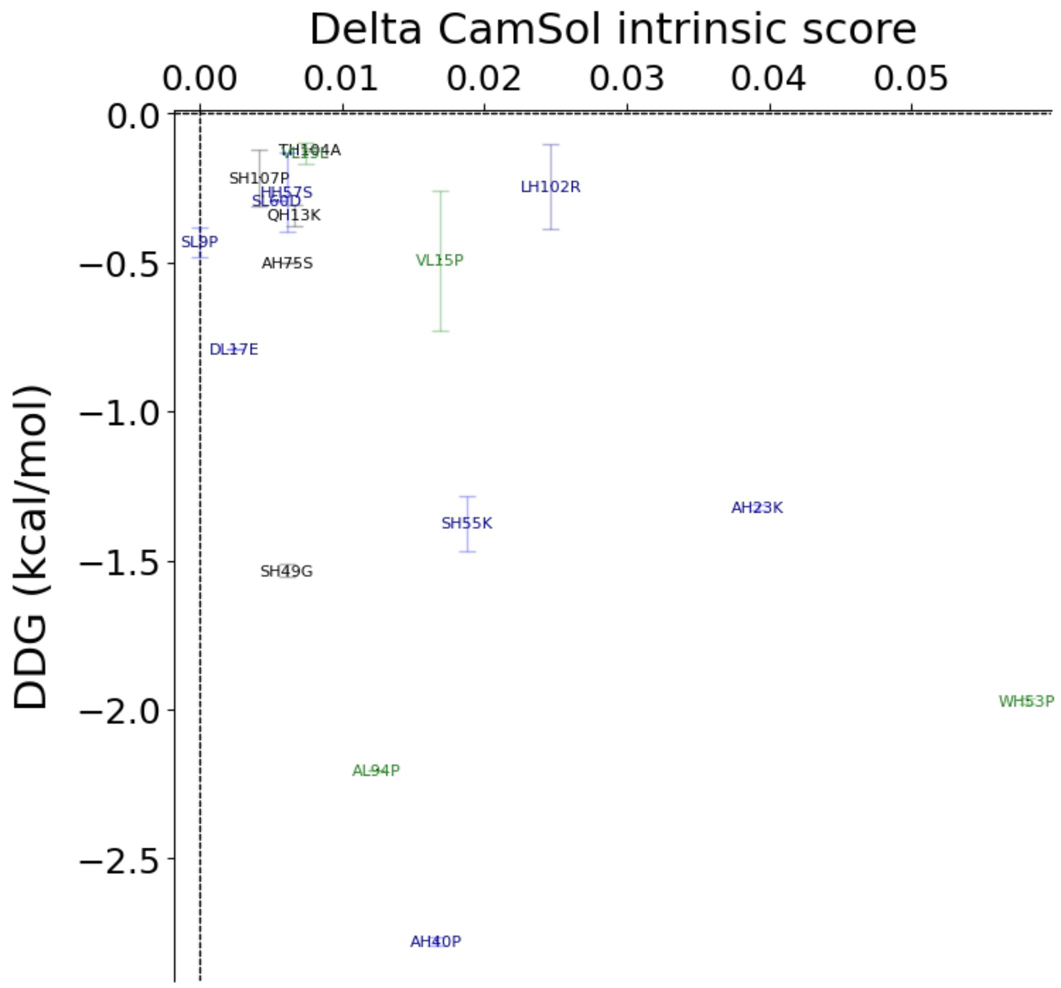

Delta CamSol intrinsic score
0.00
0.01
0.02
0.03
0.04
0.05
0.0
−0.5
−1.0
−1.5
−2.0
−2.5
DDG (kcal/mol)
TH104A
VL19E
SH107P
LH102R
HH57S
SL60D
QH13K
SL9P
AH75S
VL15P
DL17E
SH55K
AH23K
SH49G
WH53P
AL94P
AH40P

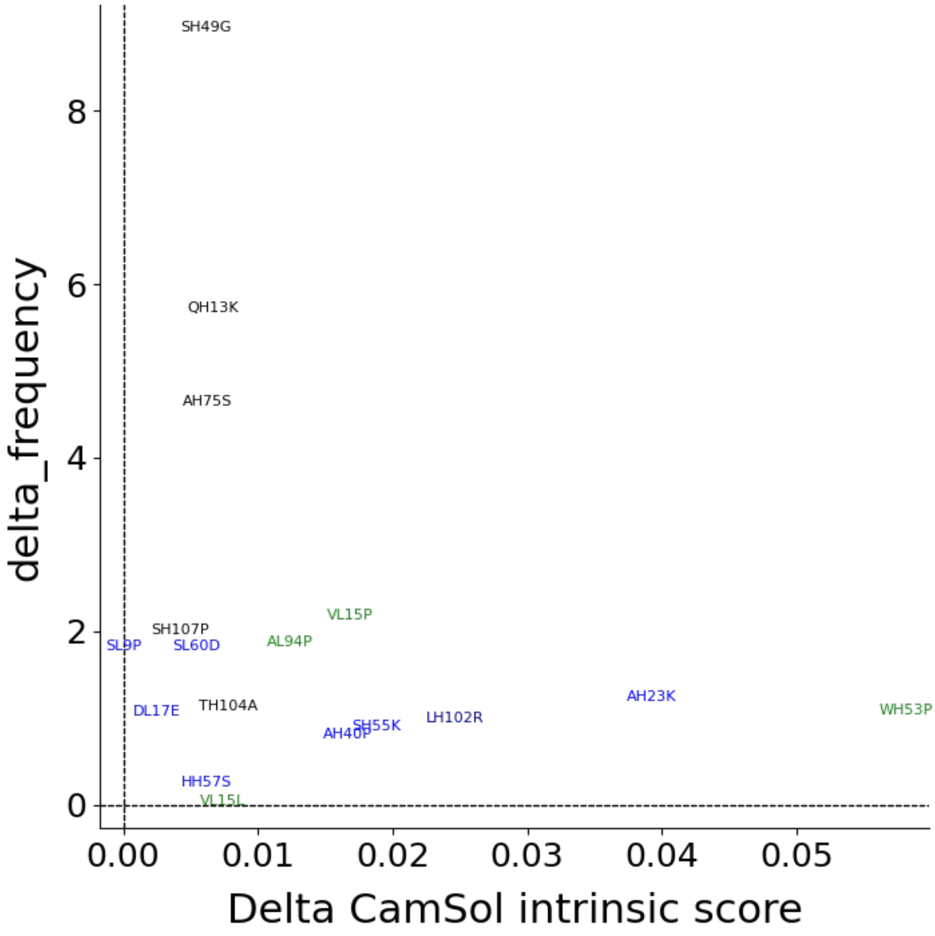

Plot of the data in the previous table, points are candidate mutations numbered according to the residue number within the input pdb file. The best mutations are those with large Delta CamSol score and large negative predicted DDG (if FoldX was run) and/or large positive Delta Frequency in MSA. Points in black correspond to mutation sites selected according to their conservation (see previous table), in green according to the sequence-based intrinsic solubility prediction, and in blue according to the structurally corrected one. Mutations at sites selected according to the solubility profiles (blue and green) are expected to have more impact on solubility than mutations at sites selected from conservation (black).

Mutation score of results of single-mutation scanning at all suitable sites (before normalisation)

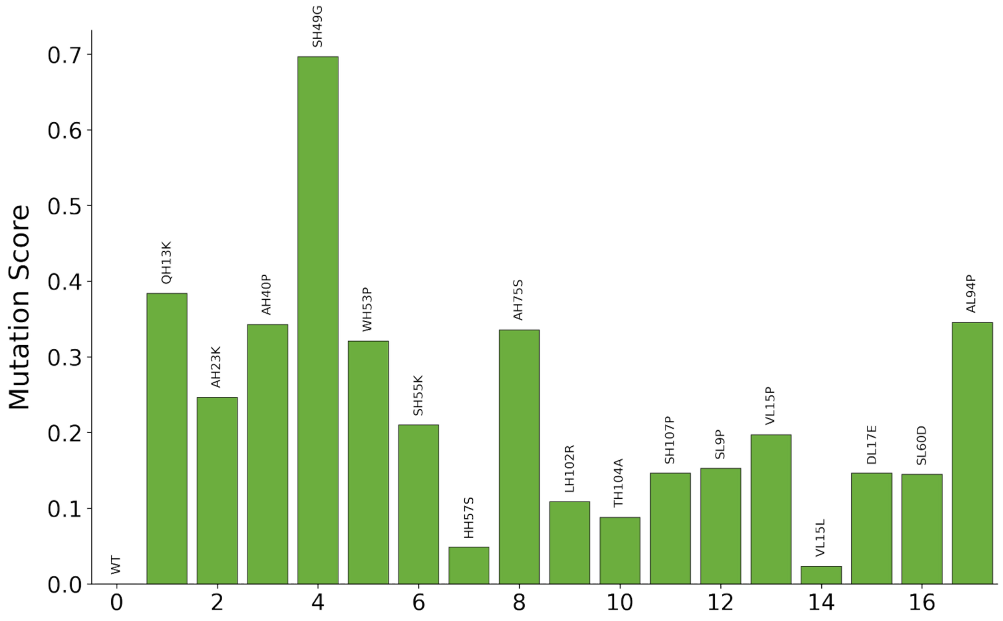

The plotted score is a rather arbitrary combination of delta solubility score, delta frequency, and predicted DDG (if FoldX was run). The highest this score the best the

mutation. This score provides a nice visual ranking of mutations, but in practice one should refer to actual delta solubility score, delta frequency, and predicted DDG values to choose suitable mutations. This is for the single mutational scanning, then for combination of mutations, especially when across multiple chains, the mutation score is normalised usually resulting in a decreased contribution of the PSSM frequency (see paper).
